# A person-centred and data-driven approach to phenotyping anorexia nervosa

**DOI:** 10.1186/s40337-026-01632-8

**Published:** 2026-05-19

**Authors:** Charlotte Bovenberg, Johanna L. Keeler, Valentina Cardi, Suman Ambwani, Rebecca Morris, Katie Rowlands, Janet Treasure

**Affiliations:** 1 Centre for Research in Eating and Weight Disorders, Department of Psychological Medicine, Institute of Psychiatry, Psychology and Neuroscience, King’s College London, London, UK; 2 Department of Child and Adolescent Psychiatry, Psychosomatics and Psychotherapy, LVR University Hospital Essen, Essen, Germany; 3 Department of Psychology, University of Padova, Padova, Italy; 4 DIS Study Abroad in Scandinavia (DK), Copenhagen, Denmark; 5 South London and Maudsley NHS Foundation Trust, Bethlem Royal Hospital, Monks Orchard Road, Beckenham, Kent BR3 3BX UK

**Keywords:** Anorexia nervosa, Severity, Longstanding, Latent profile analysis, Eating disorders, Personalised treatment, Enduring

## Abstract

**Background:**

Anorexia nervosa (AN) is a severe psychiatric disorder (ED) with high mortality, marked functional impairment, and substantial phenotypical heterogeneity. Despite extensive research, treatment outcomes remain poor, and it is unclear why some individuals improve while others follow more persistent and severe courses. Existing diagnostic subtypes show limited value in predicting illness trajectory or treatment response. This study examined the distinct AN phenotypes which emerged within a treatment-seeking sample and considered how these differ in their response to standard treatment.

**Method:**

Using data from the TRIANGLE study, latent profile analysis was used to identify phenotypic subgroups of adult patients with AN or atypical AN admitted to hospital for intensive care (*n* = 382), based on a variety of anthropometric and clinical variables. Following profile allocation, separate linear mixed model analyses (*n* = 370) examined differences between groups and over time (18 months) in depression, anxiety, and stress symptoms, work and social impairment, body mass index (BMI), and ED psychopathology.

**Results:**

A four-profile solution best fit the data. Profiles differed in illness duration and symptom severity. One profile with long illness duration (mean ± standard deviation = 9 ± 7 years) and high symptom severity, a long-duration group (21 ± 11 years) with moderate severity, and two short-duration groups (4 ± 3 and 5 ± 3 years), one with high and one with comparatively lower severity, respectively. The high-severity, shorter-duration profile showed significant improvement across all variables over the 18-month follow-up period, whereas the highest-severity, longer-duration profile showed significant improvement on all variables except ED psychopathology. Where improvements were observed in both high-severity profiles, the magnitude of change was generally greater in the longer-duration profile, except for BMI, where it was equal and work and social impairment, where the shorter-duration profile showed greater improvement. In contrast, the lower-severity profiles showed significant improvements only in BMI and work and social impairment, which were smaller in magnitude than those observed in the high-severity profiles.

**Conclusion:**

Subtyping and treatment planning for AN must recognise that prognosis is shaped by multiple interacting factors rather than any single indicator and incorporate psychological, social, and functional complexity.

**Supplementary Information:**

The online version contains supplementary material available at 10.1186/s40337-026-01632-8.

## Abstract (plain language summary)

Anorexia Nervosa (AN) is a complex mental disorder with heterogeneous symptom presentations. Recovery rates are suboptimal, and it is unclear which factors predict symptom remission in the shorter and longer term. The hypothesis that illness duration might contribute to explaining symptom severity has been proposed and has received some empirical support. However, it is becoming apparent that a more complex array of individual and clinical characteristics might better explain treatment response. In this study, we used a large dataset to examine the possibility of different, distinct presentations of AN. Using a data-driven approach, participants were grouped based on illness duration, body mass index, depression and anxiety symptoms, social and work functioning, autism traits, and eating disorder behaviours. Follow-up data were considered to examine the symptom trajectories of the different groups following standard treatment for AN. The results indicated the presence of four different groups in this dataset, defined by differences in symptom severity and illness duration. One group with long illness duration (9 years) and high severity, a very long-duration group (21 years) with moderate severity, and two shorter-duration groups with an average duration of 4 and 5 years, one with high and one with comparatively lower severity, respectively. The groups differed in their response to standard treatment; individuals with initially higher impairment showed the highest symptom improvement. The findings highlight that a single indicator, such as weight or length of illness, is not enough to subtype AN and predict prognosis. Treatment planning should consider different factors such as psychopathology, illness duration, and social impairments.

**Supplementary Information:**

The online version contains supplementary material available at 10.1186/s40337-026-01632-8.

## Background

Anorexia nervosa (AN) is a severe and complex eating disorder (ED). It is associated with profound functional impairment and has one of the highest mortality rates of all psychiatric conditions [1]. It is a heterogeneous disorder, complicated by frequent psychiatric and medical comorbidities [2]. Recovery rates vary depending on patient cohorts and treatment centres, ranging from 40% to 62.8% [3, 4]. Although several risk factors for poorer outcomes have been identified [3, 5–7], the mechanisms underlying the variability and complexity of illness trajectories remain difficult to disentangle. Low recovery rates may reflect broader structural and environmental influences, such as disparities in treatment access and healthcare systems [8–11], but also a complex array of individual and clinical characteristics that make the illness more resistant to standard treatments. A clearer understanding of these characteristics would support the development of tailored interventions for individuals with enduring difficulties and inform strategies to reduce progression toward long-term, complex presentations.

Currently, AN is subtyped in the Diagnostic and Statistical Manual of Mental Disorders, Fifth Edition (DSM-5) according to two parameters: severity, determined by body mass index (BMI), and eating disorder-specific behaviours, defined as either restricting food intake or binge-purging [12]. The clinical utility of these classifications has been widely questioned by researchers, clinicians, and individuals with lived experience [13–15]. The behavioural subtypes show limited predictive value, as individuals frequently transition between restricting and binge-purging presentations over time [16]. Evidence further indicates little difference in treatment response, relapse, or mortality across diagnostic subtypes [13, 14]. BMI-derived severity thresholds do not meaningfully differentiate individuals in terms of frequency of ED behaviours or psychological distress [15, 17]. A key limitation is that they overlook absolute weight loss, that is, the amount of weight lost from premorbid levels. Many individuals with lived experience further report that BMI fails to capture their mental state, noting that higher BMI values can coincide with greater subjective psychological distress [18–20]. This is particularly evident in atypical AN, where individuals experience high levels of distress and impairment despite not crossing DSM-5-defined BMI thresholds [21]. Therefore, while BMI has an important role as a direct and objective indicator of clinical risk and mortality [22], it should not be the sole marker of severity. Instead, it should be complemented by other indicators, such as occupational and social functioning, chronicity, psychiatric comorbidity, and symptom complexity, given their established associations with treatment outcomes and their recognition by individuals with lived experience as central features of AN [5, 20, 23, 24]. If the current DSM-5-derived classifications are limited in their clinical utility [14, 15], how might their usefulness be improved? Data-driven, empirically grounded approaches to subtyping AN that predict treatment response and inform targeted interventions may offer a solution.

In eating disorders, one clinically hypothesised, but not empirically validated, phenotype is the group described as having severe and enduring anorexia nervosa (SE-AN), estimated to comprise about 20% of individuals with a prolonged and severe illness course [25]. While originally intended to facilitate treatment tailoring, many individuals with lived experience have criticised the term as overly deterministic [18]. Consequently, the present paper adopts the more recently proposed term severe and longstanding anorexia nervosa (SL-AN) [18]. However, a previous empirical study did not identify a distinct subgroup corresponding to this construct [26]. This absence of empirical evidence showing that patient characteristics cluster into a distinct, treatment-resistant phenotype represents a substantial limitation of the SL-AN construct. Its current classification, primarily based on illness duration and prior treatment attempts [27], might further obscure important differences in functioning and clinical need between individuals with long-term AN [19]. Illness complexity (e.g., comorbidity, neurodivergence, social impairments [5, 28]) needs to be considered when conceptualising SL-AN.

More broadly, heterogeneity within AN remains poorly understood [29]. Despite substantial advances in our understanding of risk and maintenance factors [5, 30], including growing recognition of neurodiversity [28, 31], the literature has not systematically examined how these dimensions integrate with other clinical features [26]. Investigations are lacking as to whether factors such as comorbidity, neurodiversity, chronicity, and ED psychopathology cluster together to form distinct subgroups. Exploratory, data-driven approaches offer a means of identifying naturally occurring subgroups based on a wide range of relevant characteristics, rather than imposing predefined categories [26]. This is especially urgent as the absence of empirically derived classifications based on symptom constellations hinders progress towards personalised treatment [29]. In parallel, evaluating whether empirically derived subgroups show differential patterns of change during treatment may help clarify their clinical relevance beyond cross-sectional description. Addressing this gap is therefore critical to improving outcomes and equity in treatment provision.

The present study used a data-driven approach to identify potential phenotypes within AN and examined their response to standard treatment. Latent profile analysis (LPA) was applied to detect naturally occurring patterns across key clinical and functional variables. These variables were selected from a pre-existing dataset and included a range of characteristics well documented in AN and highlighted by individuals with lived experience, such as illness duration, BMI, depression, anxiety, autism spectrum quotient, work and social impairment, and ED psychopathology [18–20, 28, 30]. To assess the clinical relevance of the identified profiles, linear mixed models (LMM) were used to examine symptom trajectories following treatment. Given the exploratory nature of the study, no hypotheses were specified regarding profile structure or treatment response.

## Methods

### Participants and design

Participants (*n* = 382) included individuals from UK-based inpatient (*n* = 292) or day-patient (*n* = 90) settings recruited in the TRIANGLE study. This was a randomised controlled trial testing whether ECHOMANTRA, an online guided self-management intervention including psychoeducational workbooks, short videoclips and chat-based groups for patients and/or their carers, would improve clinical outcomes of intensive treatment when delivered in addition to treatment as usual (TAU) and compared to TAU alone [32, 33]. The design and primary outcomes of the TRIANGLE study are detailed in a separate publication [33].

Clinical staff at NHS and independent hospitals screened inpatients and day patients for eligibility. Potentially eligible patients were approached by clinical study officers or research assistants, who provided study information and obtained written informed consent. Consenting patients provided contact details for potential carers, who were then approached by the local investigator or clinical study officer to obtain consent for participation.

Participants were eligible for recruitment at any point between their first day of admission to inpatient or day-patient treatment and four weeks post-discharge. Inclusion criteria were required to be met at the time of recruitment, when informed consent was obtained. Eligible individuals were aged 16 years or older, had a DSM-5 diagnosis of AN or atypical AN and were either receiving inpatient treatment or day-patient treatment for a minimum of three days per week. Eligibility additionally required the availability of a carer willing to participate in the study, participant agreement to carer involvement, and access to an internet-enabled electronic device to use the study platform. Participants were excluded if they had inadequate English proficiency, a serious psychiatric or chronic physical health condition requiring treatment (e.g. psychosis, diabetes, or cystic fibrosis), were pregnant, or had previously participated in an intervention using ECHOMANTRA materials. The full dataset was used for the present secondary analysis; however, the sample for the longitudinal analyses consisted of fewer participants (*n* = 370), as twelve participants who completed the baseline assessment did not proceed with the trial.

### Measures

LPA was conducted using patient baseline data, while the LMMs also utilised data from 3, 6, 9, 12, and 18-month follow-ups. Sociodemographic and clinical details were self-reported at baseline [32, 33] and included psychiatric comorbidities (autism spectrum disorder, attention-deficit/hyperactivity disorder (ADHD), generalised anxiety disorder (GAD), major depressive disorder (MDD), obsessive-compulsive disorder (OCD), panic disorder), ethnicity, sex, and age. Illness duration was defined as the duration since the onset of ED symptoms and was assessed by asking the participants the following question: “For how many years have you had an eating disorder?” Age at onset was derived by subtracting illness duration from the participant’s age. BMI was calculated using clinician-reported height and participant-reported weight, as kg/m^2^. It was estimated at baseline, 3, 6, 9, 12, and 18 months. In addition, the following questionnaires were used:

#### Autism spectrum quotient-10 (AQ-10)

Traits of autism spectrum condition were assessed using the AQ-10, a 10-item self-report measure [34]. Scores reflect the mean of all items. The AQ-10 demonstrated acceptable internal consistency in this sample (Cronbach’s *α* = 0.71). It was measured at baseline.

#### Depression anxiety stress scale (DASS-21)

Symptoms of depression, anxiety, and stress were measured using the DASS-21, a 21-item self-report scale comprising three 7-item subscales [35]. Each subscale score is the sum of its items, doubled to facilitate its comparability with the original DASS. The scale demonstrated high reliability for the depression, anxiety, and stress subscales in this sample (Cronbach’s *α* = 0.92, 0.85, and 0.86, respectively). It was measured at baseline, 3, 6, 9, 12, and 18 months.

#### Eating disorder examination questionnaire (EDE-Q)

ED psychopathology was evaluated using the EDE-Q [36], which provides scores on four subscales and a global score. The global score is the average of all item responses. The EDE-Q demonstrated strong internal consistency in this sample (Cronbach’s *α* = 0.95). It was measured at baseline, 3, 6, 9, 12, and 18 months.

#### Obsessive-compulsive inventory (OCI)

Obsessive-compulsive symptoms were assessed using the OCI [37], a self-report measure evaluating distress from common OCD symptoms. Scores are calculated as the sum of all items. The OCI demonstrated high reliability in this sample (Cronbach’s *α* = 0.92). It was measured at baseline.

#### Work and social adjustment scale (WSAS)

Functional impairment was measured using the WSAS [38], a 5-item scale assessing the impact of psychological symptoms on daily functioning. Total scores are summed across items. The WSAS demonstrated good reliability (Cronbach’s *α* = 0.83) in this sample. It was measured at baseline, 6, 12, and 18 months.

### Ethical approval and consent procedures

The study was approved by the London-Camberwell St Giles Research Ethics Committee (Reference: 16/LO/1377). Written informed consent was obtained from all participants prior to the start of the trial.

### Statistical analyses

The sample’s demographic and clinical data were characterised using means and standard deviations (SD) for continuous variables and raw numbers and proportions for categorical variables. All statistical analyses were conducted using R version 4.5.1 [39] or SPSS version 29.0.2.0.

#### Latent profile analysis

##### Model estimation and selection

An LPA was conducted to identify latent subgroups (profiles) of individuals who respond similarly across multiple continuous variables, namely, illness duration (years), BMI, EDE-Q global score, WSAS total score, DASS depression score, DASS anxiety score, and AQ-10 total score. The measures were selected based on discussions within the research group, which took into account prior literature on risk and maintenance factors [5, 30, 31], qualitative reports of what individuals with lived experience consider defining features of their illness [18, 19], and input from a patient and public involvement group from a separate study, which explored what people with lived experience consider meaningful indicators of change. Together, these variables capture a broad and clinically relevant range of variables, encompassing core ED symptoms, associated psychopathology, functional impairment and markers of neurodevelopmental traits. The selection of indicators was deliberately restricted, as incorporating additional variables would have expanded the number of freely estimated class-specific parameters beyond the capacity permitted by the sample size, leading to instability and model non-convergence. All variables were standardised before the LPA. Missing data (Table SI.1) were imputed through predictive mean matching via the ‘mice’ package (SII.2).

LPA model solutions specifying between one and six profiles were estimated. Model fit was evaluated using different statistical indices such as the Bayesian Information Criterion, entropy, and the analytic hierarchy process (AHP) [40, 41]. Due to limitations in the R implementation of the Bootstrapped Likelihood Ratio Test (BLRT), the BLRT could not be run, potentially limiting statistical support for the optimal model. In addition to statistical fit, model interpretability was evaluated. The model solution offering the best statistical fit and interpretability, judged in line with existing research, was selected for further analysis [40]. Following model selection, profile membership was assigned based on the highest posterior probability per individual. Further information about model estimation and selection can be found in the supplementary material (SII.1).

##### Model evaluation and exploration

All model evaluations and explorations were conducted using the non-standardised dataset. One-way ANOVAs (or Kruskal-Wallis tests when assumptions were not met) were used to assess differences between profiles for each variable included in the LPA. Homogeneity of variances was assessed using Levene’s test, while normality was evaluated through the Kolmogorov-Smirnov test. The significance levels were Bonferroni-corrected to account for multiple comparisons and set at *α* < 0.007 (*α* = 0.05/7 tests). Effect sizes were estimated using eta squared (small: *η*^*2*^ ≥ 0.01, medium: *η*^*2*^ ≥ 0.06, large: *η*^*2*^ ≥ 0.14) [42]. Dunn’s post-hoc tests were conducted to identify specific between-profile contrasts, with Hedge’s *g* as effect sizes (small: *g* ≥ 0.20, medium: *g* ≥ 0.50, large: *g* ≥ 0.80) [42]. For the post-hoc tests, the significance levels were set to *α* < 0.05 as Dunn’s tests return multiple-comparison-corrected *p*-values.

The same statistical procedures were applied to additional exploratory continuous variables not included in the LPA: age, DASS stress scores, number of comorbidities, OCI total scores, and the mean number of days from admission to baseline assessment, to evaluate the robustness of profile distinctions and examine additional factors that may contribute to between-profile variability. Finally, chi-square tests assessed whether profiles differed by several additional categorical variables: treatment type (inpatient vs. day-patient), the proportion of participants who were currently or ever sectioned under the Mental Health Act (yes vs. no), or who reported lifetime diagnoses of MDD, GAD, autism, panic disorder, OCD, and ADHD. Effect sizes were reported using Cramer’s *V* (small: *V* < 0.30, medium: *V* ≥ 0.30, large: *V* > 0.50) [42]. Bonferroni-corrected significance levels of *α* < 0.0045 (*α* = 0.05 / 11 tests) were applied to all analyses of variables not included in the LPA.

#### Linear mixed model

LMMs were fitted using restricted maximum likelihood with Profile, Time, and their interaction (Profile x Time) as fixed effects to examine whether the latent profiles differed in their symptom trajectories and thereby their responses to standard treatment over time. Models included random intercepts and random slopes for Time to allow for individual differences in baseline levels and rates of change. Although the data were taken from a treatment trial, treatment type was not included as a covariate, as there was no significant treatment effect [32, 33]. Separate models were run for each outcome variable: total EDE-Q score, the DASS Depression, Anxiety, and Stress subscales, WSAS, and BMI. Baseline measurements and data from the 3-, 6-, 9-, 12-, and 18-month follow-up assessments were included for all variables except WSAS, which only had follow-up data available at 6, 12, and 18 months.

## Results

### Descriptive statistics

The sample was predominantly composed of women (*n* = 346 [93.5%]) and individuals identifying as white (*n* = 350 [94.9%]). The mean BMI was in the DSM-5-defined severe range (*M ± SD =* 14.51 *±* 1.85 kg/m^2^) [12]. Participants were primarily adults (16–67 years), with a wide range of illness duration (0–54 years). Mean scores on measures of symptom severity and functional impairment for the whole sample were above the population-normed “severe” threshold for each questionnaire, apart from AQ-10 [12, 34, 35, 37, 38, 43]. The majority of the sample had a lifetime diagnosis of MDD or GAD (62.5%, 59.2%, respectively), with some having both diagnoses (46.1%). Most participants were receiving inpatient care when they joined the study (76.4%), while the remainder were receiving day patient care. Participants had been admitted, on average, approximately two months before their baseline assessment (77.38 ± 76.17 days) (Table 1).

**Table 1:** Number and proportions or means and standard deviations for all variables

Variable	Total sample*	Profile 1 Higher S-Shorter D	Profile 2 Highest S-Longer D	Profile 3 Lowest S-Shorter D	Profile 4 Lower S-Longest D
n	382	147	79	98	58
Age (years), mean [SD]	25.45 [8.90]	22.17 [5.31]	25.01 [7.24]	23.33 [6.25]	37.88 [11.23]
Illness Duration (years), mean [SD]	8.08 [8.26]	4.38 [2.67]	8.94 [7.06]	4.92 [3.46]	21.32 [10.68]
BMI, mean [SD]	14.51 [1.85]	14.72 [1.73]	14.48 [1.70]	14.41 [1.95]	14.18 [2.17]
Age at Onset (years), mean [SD]	17.42 [5.90]	17.84 [5.84]	16.07 [4.66]	18.44 [5.90]	16.56 [7.16]
Sex					
Female, n (%)	346 (93.5)	131 (94.2)	73 (93.6)	89 (91.8)	53 (94.6)
Male, n (%)	24 (6.5)	8 (5.8)	5 (6.4)	8 (8.2)	3 (5.4)
Ethnicity					
Asian, n (%)	5 (1.4)	1 (0.7)	2 (2.6)	2 (2.1)	0 (0.0)
Black, n (%)	2 (0.5)	0 (0.0)	2 (2.6)	0 (0.0)	0 (0.0)
White, n (%)	350 (94.9)	132 (95.0)	72 (92.3)	91 (94.8)	55 (98.2)
Mixed, n (%)	12 (3.3)	6 (4.3)	2 (2.6)	3 (3.1)	1 (1.8)
Days since admission**, mean [SD]	77.38 [76.17]	83.22 [88.21]	69.97 [67.25]	72.06 [54.99]	81.67 [85.35]
Treatment Type					
Inpatient, n (%)	292 (76.4)	107 (72.7)	66 (83.50)	74 (75.5)	45 (77.6)
Day-patient, n (%)	90 (23.6)	40 (27.3)	13 (16.50)	24 (24.5)	13 (22.4)
AQ-10 total, mean [SD]	4.32 [2.22]	4.77 [2.03]	4.94 [0.53]	2.99 [1.77]	3.76 [2.06]
EDE-Q total, mean [SD]	3.87 [1.38]	4.25 [0.79]	5.03 [0.53]	2.56 [1.43]	3.67 [1.55]
OCI total, mean [SD]	27.86 [15.30]	29.49 [12.60]	38.54 [17.58]	18.03 [10.82]	25.70 [14.01]
WSAS total, mean [SD]	24.22 [9.20]	24.98 [7.48]	31.71 [5.91]	16.37 [8.09]	24.98 [8.87]
DASS total, mean [SD]	70.49 [27.30]	76.03 [13.23]	105.27 [10.87]	39.46 [14.49]	60.04 [22.98]
Depression, mean [SD]	26.42 [11.46]	29.64 [7.20]	39.48 [2.82]	13.81 [7.67]	20.31 [9.66]
Anxiety, mean [SD]	17.20 [10.03]	17.92 [5.75]	29.44 [7.13]	7.45 [5.30]	15.55 [9.78]
Stress, mean [SD]	26.65 [9.38]	28.47 [6.29]	36.41 [5.00]	17.54 [6.60]	23.82 [9.41]
Comorbidities n, mean [SD]	1.69 [1.33]	1.58 [1.25]	2.33 [1.21]	1.14 [1.34]	1.98 [1.28]
Diag. MDD, n (%)	230 (62.5)	84 (60.9)	64 (83.1)	36 (37.1)	46 (82.1)
Diag. Anxiety, n (%)	218 (59.2)	81 (58.7)	59 (76.6)	39 (40.2)	39 (69.6)
Diag. OCD, n (%)	60 (16.3)	22 (15.9)	19 (24.7)	11 (11.3)	8 (14.3)
Diag. ADHD, n (%)	9 (2.4)	5 (3.6)	1 (1.3)	2 (2.1)	1 (1.8)
Diag. ASD, n (%)	20 (5.4)	9 (6.5)	7 (9.1)	2 (2.1)	2 (3.6)
Diag. PD, n (%)	23 (6.2)	7 (5.1)	7 (9.1)	2 (2.1)	7 (12.5)

The profiles displayed here (Higher Severity-Shorter Duration, Highest Severity-Longer Duration, Lowest Severity-Shorter Duration, Lower Severity-Longest Duration) were estimated by LPA and denote different combinations of illness duration and symptom severity

*ADHD* Attention-Deficit/Hyperactivity Disorder, *AQ-10* Autism Spectrum Quotient − 10, *ASD* Autism Spectrum Disorder, *BMI* Body Mass Index, *D* Duration, *DASS* Depression Anxiety Stress Scale, *Diag*. = Lifetime Diagnosis, *EDE-Q* Eating Disorder Examination Questionnaire, *MDD* Major Depressive Disorder, *OCD* Obsessive Compulsive Disorder, *OCI* Obsessive Compulsive Index, *n* number of, *PD* Panic Disorder, *S* Symptoms, *SD* Standard Deviation, *WSAS* Work and Social Adjustment Scale

*Not all participants completed all baseline measurements

**Days since admission refers to the number of days that passed between admission to treatment and baseline measurement

### Latent profile analysis

#### Model choice

**Fig. 1:**
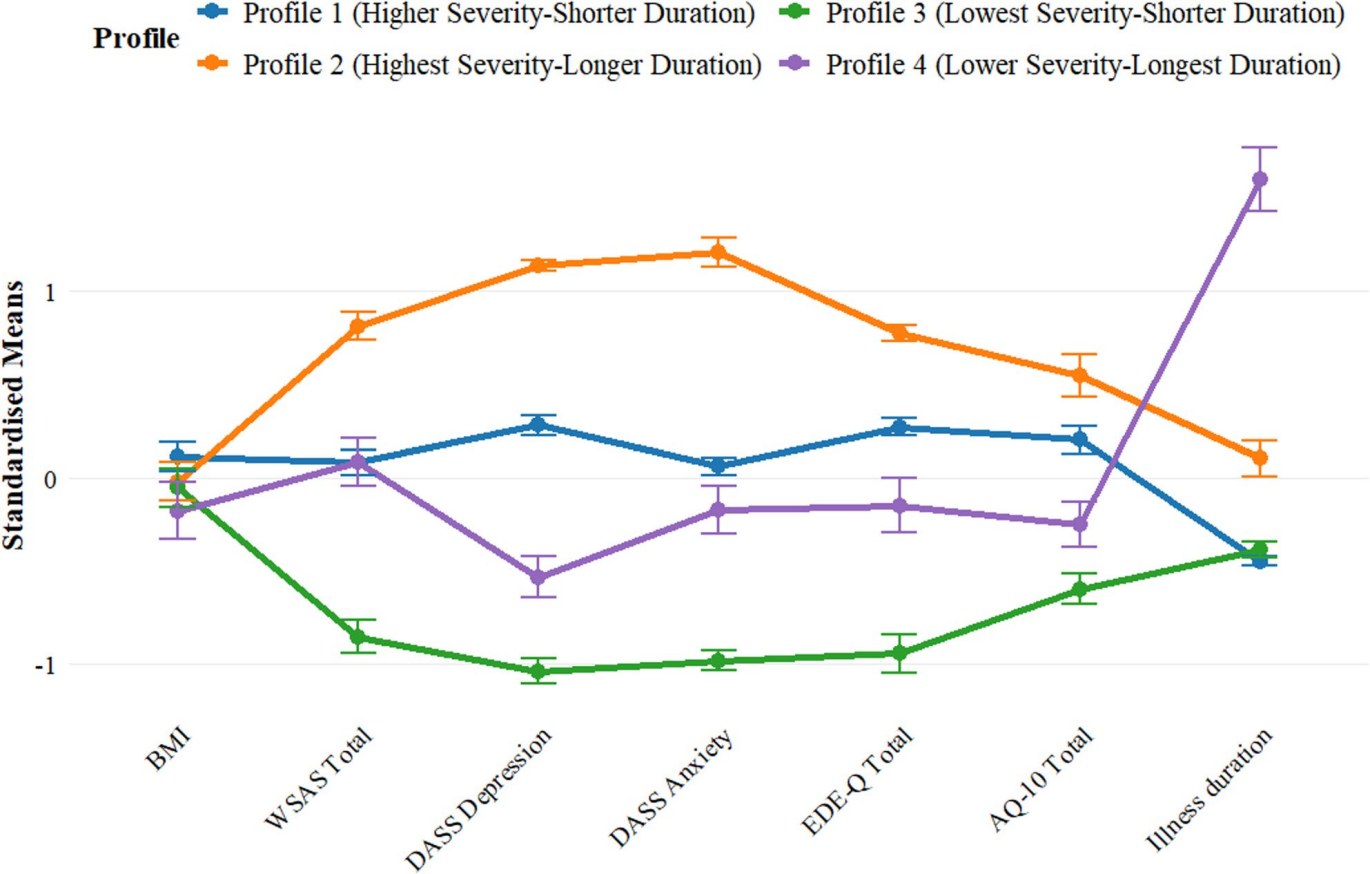
Line Graph of the Standardised Means of the Profile Solution. This line graph displays the standardised means of each profile across the variables included in the latent profile analysis. The values shown represent standardised (relative) means rather than raw means, allowing for direct comparison between profiles. The lines connecting the points are included purely for visual clarity to highlight patterns and differences between profiles and should not be interpreted as indicating a progression over time or any causal relationship. The profiles displayed here (*Higher Severity-Shorter Duration* (blue), *Highest Severity-Longer Duration* (orange), *Lowest Severity-Shorter Duration* (green), *Lower Severity-Longest Duration* (purple)) denote different combinations of illness duration and symptom severity. Error bars represent standard error. *AQ-10* Autism Spectrum Quotient-10, *DASS* Depression Anxiety Stress Scale, *EDE-Q* Eating Disorder Examination Questionnaire, *WSAS* Work and Social Adjustment Scale

The independent model fit indices did not support a single best-fitting solution (Table SI.2). The AHP [41], taking into account several fit indices, identified the four-profile model as the most suitable overall. Accordingly, subsequent analyses focused on the interpretability and distinctiveness of the four-profile solution (Fig. 1).

The LPA was conducted using standardised scores to prevent variables with larger ranges from disproportionately influencing profile formation. Lower scores reflect relatively lower values compared to other profiles, rather than indicating clinically low levels on the original measures. Severity thresholds of the variables are included in Table 2 to facilitate interpretation.

**Fig. 2:**
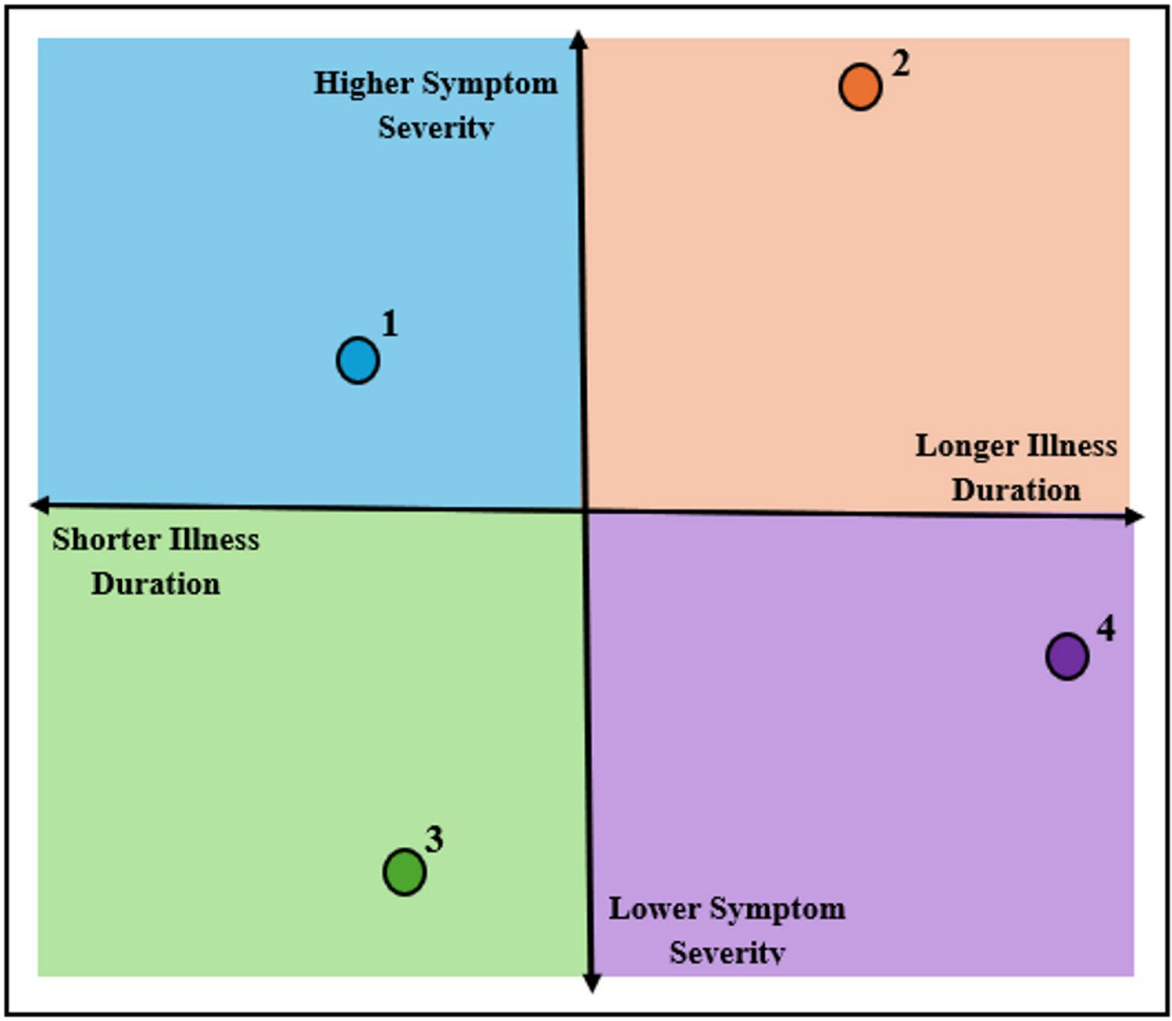
Visualisation of where the Profiles are situated in Dimensions of Symptom Severity and Illness Duration. 1 = Profile 1 (*Higher Severity-Shorter Duration*), 2 = Profile 2 (*Highest Severity-Longer Duration*), 3 = Profile 3 (*Lowest Severity-Shorter Duration*), 4 = Profile 4 (*Lower Severity-Longest Duration*)

The selected four-profile model (Fig. 1) identified distinct profiles based on combinations of illness duration and symptom severity. Profile names were derived from comparative levels of symptoms and lengths of illness duration. They are entirely contextual and should be interpreted relative to this high-severity, clinical sample. Profile 1 (*Higher Severity-Shorter Duration*; *n* = 147, $\hat{p}$ = 38.5%) was characterised by a comparatively shorter illness duration (*Mean* = 4.38 years, *SD* [2.67]) and higher symptom severity. Profile 2 (*Highest Severity-Longer Duration*; *n* = 79, $\hat{p}$ = 20.7%) showed a longer illness duration (8.94 years [7.06]) with consistently very high to extreme symptomatic severity. Profile 3 (*Lowest Severity-Shorter Duration*; *n* = 98, $\hat{p}$ = 25.6%) presented with a shorter illness duration (4.92 years [3.46] and the comparatively lowest symptom severity. Profile 4 (*Lower Severity-Longest Duration*; *n* = 58, $\hat{p}$ = 15.2%) was marked by very long illness duration (21.32 years [10.68]) and lower symptoms. These results indicate meaningful differentiation among profiles based on symptom severity and illness duration, supporting the four-profile model’s validity (Fig. 2).

**Table 2:** Mean Scores ± SDs on the severity variables per profile compared to literature-derived thresholds of “severe”

Variable	Higher S-Shorter D	Highest S-Longer D	Lowest S-Shorter D	Lower S-Longest D	Literature-derived Thresholds			
					Mild	Moderate	Severe	Extreme
AQ-10	4.77 ± 2.03	5.53 ± 0.53	2.99 ± 1.77	3.76 ± 2.06	NA	NA	≥ 6**	NA
BMI	**14.72** ± 1.73	**14.47** ± 1.70	**14.41** ± 1.95	**14.18** ± 2.17	≥ 17.0	16-16.99	15-15.99	< 15
EDE-Q	**4.24** ± 0.79	**5.00** ± 0.53	2.56 ± 1.43	3.68 ± 1.55	NA	NA	4	NA
Depression	**29.64** ± 7.20	**39.48** ± 2.82	13.81 ± 7.67	20.31 ± 9.66	10–13	14–20	21–27	> 27
Anxiety	**17.92** ± 5.75	**29.44** ± 7.13	7.45 ± 5.30	**15.55** ± 9.78	8–9	10–14	15–19	> 19
Stress	**28.47** ± 6.29	**36.41** ± 5.00	17.54 ± 6.60	25.70 ± 9.41	15–18	19–25	26–33	> 33
OCI	**29.48** ± 12.60	**38.54** ± 17.58	18.03 ± 10.82	**23.82** ± 14.01	NA	NA	≥ 21*	NA
WSAS	**24.98** ± 7.48	**31.71** ± 5.91	16.37 ± 8.09	**24.98** ± 8.87	< 9	10–20	> 20	NA

Mean scores that cross the “severe” threshold are printed in bold. Depression, Anxiety, and Stress refer to the corresponding DASS subscales

*AQ-10* Autism Spectrum Quotient − 10, *BMI* Body Mass Index, *D* Duration, *DASS* Depression Anxiety Stress Scale, *EDE-Q* Eating Disorder Examination Questionnaire, *NA* Not Applicable, *OCI* Obsessive Compulsive Inventory, *S* Symptoms, *SD* Standard Deviation, *WSAS* Work and Social Adjustment Scale

*An OCI cut-off of 21 is deemed best to differentiate between people with obsessive-compulsive disorder and those without [37].

**A score of 6 or higher indicates possible autism according to the NICE guidelines. Cut-off definitions: BMI [12], DASS subscales [35], EDE-Q [43], WSAS [38]

#### Model validation

Group comparisons revealed that the profile differences depicted by the LPA were significant across AQ-10 (*H*(3) = 67.6, *p* < 0.001, *η*^*2*^ = 0.17), DASS Depression (*H*(3) = 235.9, *p* < 0.001, *η*^*2*^ = 0.62) and Anxiety (*H*(3) = 209.41, *p* < 0.001, *η*^*2*^ = 0.55), EDE-Q (*H*(3) = 128.9, *p* < 0.001, *η*^*2*^ = 0.34), and WSAS (*H*(3) = 121.46, *p* < 0.001, *η*^*2*^ = 0.31). The overall pattern was consistent, with the *Highest Severity-Longer Duration* profile exhibiting the highest severity levels on the WSAS (*M ± SD* = 31.71 [5.91]), EDE-Q (4.94 [0.53]), DASS Depression (39.48 [2.82]) and Anxiety (29.44 [7.13]), but having a comparable score to *Highest Severity-Longer Duration* on the AQ-10 (5.53 [2.25]; 4.77 [2.03]). *Higher Severity-Shorter Duration* scored significantly higher than *Lower Severity-Longest Duration* on DASS Depression (29.64 [7.20]; 20.31 [9.66]), EDE-Q (4.25 [0.79]); (3.67 [1.55]), and AQ-10 (4.77 [2.03]; 3.76 [2.06]), but showed comparable scores to *Lower Severity-Longest Duration* on WSAS (24.98 [7.48]; 24.98 [8.87]) and DASS Anxiety (17.92 [5.74]; 15.55 [9.78]). *Lowest Severity-Shorter Duration* consistently reported the lowest scores across most variables, differing significantly from all other profiles except on the AQ-10 (2.99 [1.77]), where no significant difference from *Lower Severity-Longest Duration* was observed. The only notable exceptions to these patterns were illness duration (*H*(3) = 141.79, *p* < 0.001, *η*^*2*^ = 0.38), as previously described, and BMI, which did not significantly differ between profiles (*H*(3) = 4.75, *p* = 0.191, *η*^*2*^ = 0.01). Full post-hoc results can be found in Supplementary Material I (Table SI.3).

#### Model exploration

Model validation was extended to variables not included in the latent profile analysis (Figure S1). Kruskal-Wallis test results, revealed significant age differences (*H*(3) = 96.64, *p* < 0.001, *η*^*2*^ = 0.25): *Lower Severity-Longest Duration* had a significantly higher mean age than all other profiles (*Mean* = 37.88, *SD* [11.23]), whereas *Highest Severity-Longer Duration* (25.01 [7.24]) only had a higher mean age than *Higher Severity-Shorter Duration* (22.16 [5.32]), no other age differences were significant. There was no significant difference between profiles regarding the time between them being admitted to in- or day-patient care and their baseline measurements (*H*(3) = 2.95, *p* = 0.40, *η*^*2*^ = 0), the proportion of participants receiving day- or inpatient care (*χ²* = 3.39, *p* = 0.36), or the proportion of participants who were currently (*χ²* = 2.76, *p* = 0.43) or ever (*χ²* = 6.26, *p* = 0.10) sectioned under the Mental Health Act. Group differences were found for the DASS Stress subscale (*H(*(3) = 186.6, *p* < 0.001, *η*^*2*^ = 0.49) and the OCI (*H*(3) = 77.1, *p* < 0.001, *η*^*2*^ = 0.20). *Highest Severity-Longer Duration* exhibited the highest scores on the DASS Stress subscale (36.41 [5.00]) and the OCI total score (38.54 [17.58]), while *Lowest Severity-Shorter Duration* reported the lowest (17.54 [6.60]; 18.03 [10.82], respectively). Full post-hoc results can be found in Supplementary Material I (Table SI.4).

The number of lifetime psychiatric comorbidities also varied significantly by Profile (*H*(3) = 42.31, *p* < 0.001, *η*^*2*^ = 0.10). *Lowest Severity-Shorter Duration* (1.14 [1.34]) reported the fewest comorbidities, whereas *Highest Severity-Longer Duration* (2.33 [1.21]) and *Lower Severity-Longest Duration* (1.98 [1.28]) had the highest prevalence and did not differ significantly from each other. No significant group differences were found for lifetime diagnoses of OCD (*χ²* = 5.89, *p* = 0.12), ADHD (*χ²* = 1.39, *p* = 0.71), autism (*χ²* = 4.85, *p* = 0.18), or panic disorder (*χ²* = 8.02, *p* = 0.05). However, significant differences were observed for a lifetime diagnosis of MDD (*χ²* = 50.01, *p* < 0.001, *V* = 0.37) and GAD (*χ²* = 26.72, *p* < 0.001, *V* = 0.27). For MDD, standardised residual correlations indicated that *Highest Severity-Longer Duration* and *Lower Severity-Longest Duration* had fewer individuals without the diagnosis than expected; however, only *Highest Severity-Longer Duration* showed a significantly higher-than-expected proportion of individuals with MDD. Conversely, *Lowest Severity-Shorter Duration* had significantly more individuals without, and fewer with, a diagnosis than expected (SII.2, Figure SII.2.1). For GAD, *Highest Severity-Longer Duration* again exhibited a significantly higher-than-expected proportion of individuals with the diagnosis, and fewer without, while *Lowest Severity-Shorter Duration* showed the opposite pattern, significantly fewer individuals with GAD and more without than expected (SII.2, Figure SII.2.2).

### Linear mixed models analysis analyses

**Fig. 3:**
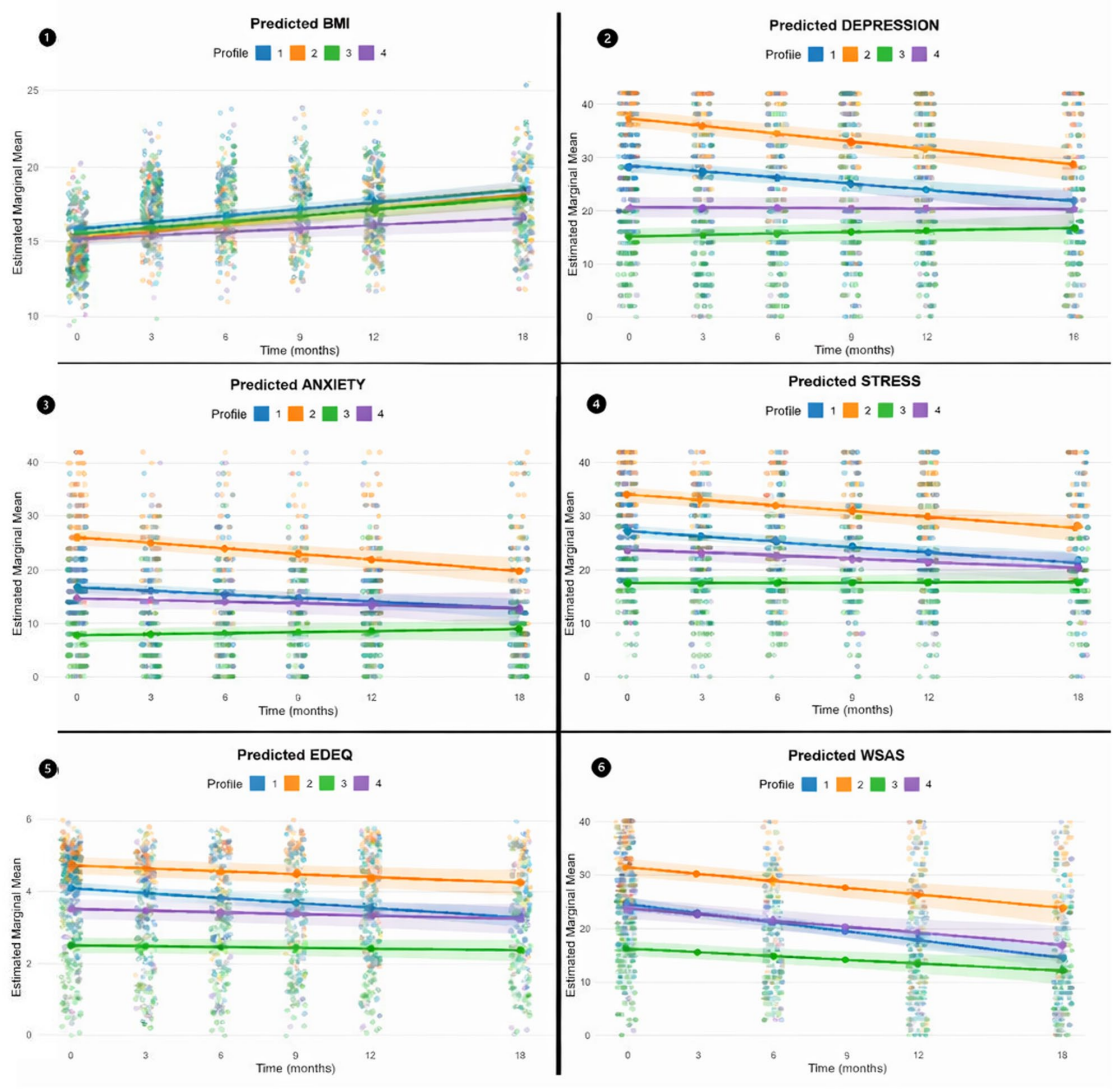
Visual Depictions of the estimated Linear Mixed Models. Profile 1 (blue) = *Higher Severity-Shorter Duration*, Profile 2 (orange) = *Highest Severity-Longer Duration*, Profile 3 (green) = *Lowest Severity-Shorter Duration*, Profile 4 (purple) = *Lower Severity-Longest Duration*. Follow-up data of the WSAS were only collected at 6, 12, and 18 months. (1) BMI, (2) DASS Depression, (3) DASS Anxiety, (4) DASS Stress, (5) EDE-Q, (6) WSAS. *BMI* Body Mass Index, *DASS* Depression Anxiety Stress Scale, *EDE-Q* Eating Disorder Examination Questionnaire, *WSAS* Work and Social Adjustment Scale

Linear mixed models (LMMs) were conducted for each outcome variable (BMI, DASS Depression, Anxiety, and Stress, EDE-Q, and WSAS) to examine differences in symptom trajectories across 18 months between the four latent profiles (Figs. 3, SI.2, SI.3; Tables SI.5-SI.9).

For BMI, only the main effect of Time was significant (*F*(1, 1157.99) = 182.84, *p* < 0.001) The estimated slopes suggest modest increases across all profiles: *Higher Severity-Shorter Duration* (*B* = 0.14, 95% *CI* [0.11, 0.17], *p* < 0.001), *Highest Severity-Longer Duration* (*B* = 0.14, [0.10, 0.18], *p* < 0.001), *Lowest Severity-Shorter Duration* (*B* = 0.13, [0.09, 0.16], *p* < 0.001), and *Lower Severity-Longest Duration* (*B* = 0.08, [0.04, 0.12], *p* < 0.001).

For the DASS Depression subscale, significant effects were found for the Profile x Time interaction (*F*(3, 293.73) = 10.40, *p* < 0.001), as well as Time (*F*(1, 293.59) = 18.89, *p* < 0.001), and Profile (*F*(3, 376.71) = 117.66, *p* < 0.001). The individual slopes show that depressive symptoms significantly decreased over time in the *Higher Severity-Shorter Duration* (*B* = -0.35, 95% *CI* [-0.47, -0.22], *p* < 0.001) and *Highest Severity-Longer Duration* (*B* = -0.49, [-0.67, -0.31], *p* < 0.001) profiles.

For the DASS Anxiety subscale, a significant Profile x Time interaction (*F*(3, 292.34) = 7.03, *p* < 0.001). was observed, along with significant main effects of Time (*F*(1, 291.92) = 19.22, *p* < 0.001) and Profile (*F*(3, 373.86) = 109.55, *p* < 0.001). The pattern mimics that of the Depression subscale, with anxiety only decreasing significantly over time in the *Higher Severity-Shorter Duration* (*B* = -0.22, 95% *CI* [-0.33, -0.11], *p* < 0.001) and *Highest Severity-Longer Duration* (*B* = -0.36, [-0.51, -0.22], *p* < 0.001) profiles.

For the DASS Stress subscale, a significant Profile x Time interaction (*F*(3, 287.37) = 4.99, *p* = 0.002), along with significant main effects for Time (*F*(1, 286.97) = 24.47, *p* < 0.001) and Profile (*F*(3, 376.81) = 100.84, *p* < 0.001) were observed. Stress decreased significantly over time in the *Higher Severity-Shorter Duration* (*B* = -0.29, 95% *CI* [-0.40, -0.18], *p* < 0.001) and *Highest Severity-Longer Duration* (*B* = -0.33, [-0.48, -0.17], *p* < 0.001) profiles.

For EDE-Q, a significant Profile x Time interaction (*F*(3, 265.01) = 4.37, *p* = 0.005), and significant main effects of Time (*F*(1, 264.49) = 27.23, *p* < 0.001) and Profile (*F*(3, 378.25) = 65.33, *p* < 0.001) were observed. EDE-Q scores decreased significantly over time in the *Higher Severity-Shorter Duration* (*B* = − 0.05, 95% *CI* [–0.06, − 0.03]), *p* < 0.001), profile.

For WSAS, a significant Profile x Time interaction (*F*(3, 271.63) = 3.60, *p* = 0.01) along with significant main effects of Time (*F*(1, 272.03) = 85.13, *p* < 0.001) and Profile (*F*(3, 380.90) = 62.35, *p* < 0.001) were found. Functional impairment decreased significantly over time in all four profiles, however the rate of change differed, with the *Higher Severity-Shorter Duration* profile (*B* = -0.56, 95% *CI* [-0.69, -0.42], *p* < 0.001) showing the largest rate of improvement followed by *Highest Severity-Longer Duration* (*B* = -0.41, [-0.60, -0.23], *p* < 0.001), *Lowest Severity-Shorter Duration* (*B* = -0.23, [-0.38, -0.08], *p* = 0.003), and *Lower Severity-Longest Duration* (*B* = -0.38, [-0.58, -0.17], *p* < 0.001) profiles.

## Discussion

In this study, an LPA revealed four distinct subgroups of individuals with AN admitted to hospital care, differentiated by illness duration and symptom severity. These findings underscore that neither dimension alone is sufficient to define clinically meaningful anorexia nervosa phenotypes.

The *Highest Severity-Longer Duration* profile (mean of 8.94 years) did not significantly differ in lifetime psychiatric comorbidity from the *Lower Severity-Longest Duration* profile (mean of 21.32 years) but otherwise exhibited the highest psychopathology and functional impairment. This profile aligns with prior characterisations of SL-AN [25, 29, 44], providing the first evidence for it being an empirically grounded clinical subgroup. This is in contrast to a previous exploratory study [26], which could be due to our inclusion of transdiagnostic features, such as neurodivergence and psychiatric comorbidities, that have been identified as being central to SL-AN in recent research [5, 28].

The *Lower Severity-Longest Duration* profile, despite its very long illness duration, showed comparatively lower acute symptom severity yet substantial social and functional impairment and very low BMI. This pattern aligns with prior literature identifying social isolation as a key factor in the maintenance of long-term AN [5], underscoring the importance of addressing psychosocial impairment during treatment [45].

The *Higher Severity-Shorter Duration* profile showed elevated psychiatric comorbidity and symptom severity across the same domains as the *Highest Severity-Longer Duration* profile, though at lower levels overall, and was distinguished by comparatively lower social and functional impairment, likely reflecting an earlier stage of illness with partially preserved social structures [30]. The *Lowest Severity-Shorter Duration* profile, while not differing from the *Higher Severity-Shorter Duration* profile in illness duration, appeared relatively less impaired compared to all other profiles.

The differences between profiles indicate that one dimension (e.g. illness duration or symptom severity) is not enough to characterise AN phenotypes or predict treatment response. This seems especially relevant considering current conversations around SL-AN, which is often defined purely based on illness duration or illness duration and prior treatment attempts [25, 27]. Symptom severity stratifying individuals with long-term AN shows that duration alone fails to capture SL-AN’s complexity. Instead, SL-AN should be viewed as a multidimensional condition involving illness duration and overlapping psychological, functional, and social impairments that perpetuate chronicity and complicate treatment.

BMI did not differ significantly between subgroups, potentially due to the small variability in the sample. Mean autism quotient scores were below diagnostic thresholds across all profiles. This contrasts with broader research indicating higher autism rates among individuals with AN [31] and may be explained by the low incidence of an autism diagnosis (5%) in the sample.

The substantial overlap in illness duration between profiles suggests that, for concepts such as SL-AN, rigid temporal cut-offs may have limited clinical utility. As profiles are characterised by patterns across multiple variables rather than illness duration alone, overlap on a single dimension does not imply equivalence between profiles. Categorising individuals as meeting criteria for SL-AN, or for another identified profile, is therefore likely to be most meaningful when guided by a formulation-based approach that integrates clinician expertise with patients’ lived experience, rather than on strict cut-offs of individual variables such as illness duration.

### Differences in symptom trajectories and treatment response

Longitudinal analyses revealed distinct patterns of change across profiles in response to standard treatment. Profiles with higher baseline symptom severity showed larger absolute reductions over time, which may partly reflect greater scope for change associated with higher initial impairment. These findings should therefore be interpreted considering baseline severity and measurement constraints.

The *Higher Severity-Shorter Duration* profile showed marked improvement across weight and psychological domains, indicating meaningful benefit from standard treatment, even allowing for potential ceiling effects. In contrast, the *Lowest Severity-Shorter Duration* profile showed limited improvement, with gains confined to BMI and work and social impairment, domains in which baseline impairment was moderate to severe. Little change was observed in domains with mild baseline impairment (depression, anxiety, stress, and ED psychopathology), consistent with floor effects or reduced standard treatment-related psychological change at lower baseline symptom severity.

The *Highest Severity-Longer Duration* profile improved substantially but remained the most impaired at 18 months, with severe scores on all measures except BMI. While weight restoration was achieved, psychological improvement was limited, suggesting that standard treatment may be insufficient to fully address the severe psychopathology and comorbidities characteristic of this group, particularly in the context of longer illness duration.

The Lower *Severity-Longest Duration* profile showed minimal change overall; while floor effects may have constrained detectable improvement, the pattern suggests limited benefit from standard care alone in the context of very long illness duration, highlighting the potential need for personalised approaches targeting illness entrenchment and identity fusion [46, 47].

Across profiles, standard treatment was effective for weight restoration, with all groups falling within the mild or moderate DSM-5 BMI range at follow-up [12]. However, most profiles, particularly the *Highest Severity-Longer Duration* profile, continued to show substantial psychopathology and functional impairment, reinforcing evidence that BMI improvement does not equate to psychological recovery [17–19].

### Clinical and research implications

Currently, care quality varies widely, particularly for long-term AN, for which no formal guidelines exist [18]. Many individuals report cycles of ineffective treatment characterised by admissions focused on weight restoration, followed by discharge and rapid relapse [18], as a defining feature of their illness [20]. Contemporary treatments for AN, such as MANTRA or multi-modal enhanced cognitive behavioural therapy, already extend beyond weight restoration to target cognitive, behavioural, and interpersonal factors. By considering markers that distinguish each profile, clinicians can more effectively target the specific comorbidities and trait patterns characteristic of those subtypes. The current findings highlight the need for strategies to treat psychiatric comorbidities among individuals with the *Highest Severity–Longer Duration* profile, with particular attention to MDD. The limited efficacy of existing pharmacological options in individuals with AN [48] suggests that novel approaches such as ketamine [49], psilocybin [50], and repetitive transcranial magnetic stimulation [51] warrant further investigation.

Anxiety, stress, and obsessive-compulsive traits also need to be addressed in both higher severity profiles and the *Lower Severity-Longest Duration* profile. Pharmacological and behavioural treatments used to treat OCD, such as exposure and response prevention, may offer promise [5, 52, 53]. In addition, improved delivery of and access to existing NICE-recommended treatment methods should be prioritised [8]. This is especially pressing for both longer-term profiles, given reports from individuals with lived experience of long-term AN, who frequently report being perceived as unlikely to benefit from treatment or, in some cases, are effectively denied access to care [11, 18].

Markers of obsessive-compulsive traits, social and work impairment, and psychiatric comorbidities could be critical early intervention targets, as they were severe in both long-term profiles, linking them to chronicity. These findings align with the literature on established risk factors and the Cognitive Interpersonal Model of SL-AN, which posits social dysfunction and comorbidity as central risk and maintaining factors of long-term AN [5, 54, 55].

In addition to its clinical relevance, the present findings have important implications for future research. Data-driven exploratory approaches rely on large datasets, as profile estimates become more reliable with increasing sample size, and larger datasets allow for the inclusion of a broader range of predictors. This highlights the need for routine, standardised clinical data collection within eating disorder services to enable replication and extension of this work. Initiatives such as the UK Eating Disorders Clinical Research Network represent an important step towards supporting larger, more robust datasets for future research.

### Strengths and limitations

A central strength of this study lies in its exploratory and longitudinal design. Although SL-AN is widely observed in clinical settings, much of the existing research has proceeded from the assumption of its existence, relying on definitions that are based on expert opinion and inconsistently applied across studies [23, 27]. LPA allows the data to determine whether subgroups emerge naturally, whereby they are not presumed but evidenced through naturally occurring data patterns. Variable selection drew on established dimensions of AN and insights from individuals with lived experience [18–20, 28, 30]. The availability of long-term follow-up data enabled examination of differences in symptom trajectories and assessment of the clinical value of the estimated subgroups. These methodological strengths may explain why the present study is the first to offer robust empirical support for an SL-AN phenotype.

Several limitations should be noted. The sample consisted only of inpatients and day-patients, representing the most severe cases of AN. While this limits generalisability, the clear differentiation between profiles even within a high-severity sample suggests similar patterns may emerge in the broader AN population. Additionally, non-female gender identities and ethnic minorities were underrepresented, reflecting broader disparities in care [56]. The relatively low entropy of the LPA model (*e* = 0.78; a minimum of 0.80 is preferred [57]) indicates some uncertainty in profile assignment; however, the high posterior probabilities and other statistical indices support the robustness of the four-profile solution.

Another limitation concerns treatment history, as the number of prior treatment attempts is an often-used indicator when characterising long-term anorexia nervosa. The TRIANGLE dataset lacked full treatment histories, preventing inclusion of this factor. However, even if such data were available, individuals with lived experience have questioned the usefulness of this dimension, arguing that it can imply personal blame and assumes that effective treatments exist, an assumption that does not hold for AN [18, 20, 25]. Further, given the absence of consensus on what constitutes a meaningful treatment attempt [44], alternative indicators, such as compulsory treatment under the Mental Health Act, were incorporated.

Finally, there are significant differences in initial symptom severity between the four profiles; this is unavoidable, as these differences are inherent to their conceptualisations. However, these differences need to be considered when discussing treatment trajectories. The observed variations might have been influenced by floor and ceiling effects, as some profiles had a greater capacity for change due to higher initial symptom severity.

### Future research directions

This study has made a first attempt at phenotyping individuals with AN based on symptom severity and illness duration. Future research should refine the definition of severity, incorporating not only symptomatology and impairment but also entrenchment, where identity becomes fused with the disorder, a phenomenon often described by those with long-term AN [18, 20]. Given that greater illness entrenchment is associated with increased difficulty imagining an AN-free identity, addressing this factor may be particularly relevant for individuals with long-term AN [58].

The present study also primarily focused on psychopathology and functional impairment; therefore, future studies should investigate potential biomarkers like pro-inflammatory cytokines [59], leptin [60], neurotrophins [60], and neuronal damage markers [61], to examine whether there are biological or cognitive differences between SL-AN and other AN subgroups. Existing studies have already linked grey matter volume loss to illness duration [62], however, the evidence for individuals with long-term AN having more pronounced memory problems and cognitive deficiencies, potential maintenance factors [63], is mixed [46, 64, 65]. Importantly, longitudinal research is needed to determine whether characteristic differences predate SL-AN in individuals with AN or arise due to long-term illness. Additionally, the present findings should be regarded as a starting point necessitating replication in other populations, and importantly, more diverse samples, encompassing individuals not in active treatment and from varied backgrounds. Further research is needed into older age groups, as they remain understudied, despite potentially distinct clinical needs and recovery trajectories compared to younger cohorts [66].

Finally, the SL-AN concept remains controversial. Critics argue that it risks implying an intrinsic or irreversible illness, potentially fostering treatment nihilism [18, 19]. Importantly, there is not enough evidence to support distinct biological or cognitive mechanisms underlying SL-AN [65]. It should further be noted that long-term recovery is possible even after an illness duration of 20 years or more [3]. Clinicians and researchers must discuss SL-AN in a manner that acknowledges the long-term nature of the illness without reinforcing the assumption of inherent or irreversible pathology. Language and framing should support patient engagement and hope, as overly rigid classifications or pessimistic discourse may unintentionally contribute to disengagement and hinder access to effective care [18]. Incorporating age thresholds into definitions of SL-AN may help avoid prematurely labelling adolescents, consistent with DSM-5 approaches to other chronic psychiatric disorders [12].

## Conclusion

This study highlights heterogeneity within AN and exemplifies possible subtypes based on differences in illness duration and symptom severity. Differences in standard treatment response across profiles highlight the need for tailored approaches for some subgroups. Social problems may be key to address, as they have been linked to poorer outcomes and were elevated in both long-duration profiles, indicating a link with chronicity. The findings reinforce that subtyping and treatment planning for AN must incorporate psychological, social, and functional complexity and that BMI is an inadequate marker of illness severity on its own. In contrast to a previous study, it provides empirical evidence that SL-AN represents a clinically meaningful group, emphasising that illness duration is not sufficient to characterise it, as symptom severity further stratifies individuals with long-term AN.

## Electronic Supplementary Material

Below is the link to the electronic supplementary material.

Supplementary Material 1.

Supplementary Material 2.

## Data Availability

The data supporting the findings of this study are available on reasonable request from VC and JT.

## Abbreviations

ADHD: Attention-deficit/hyperactivity disorder
AHP: Analytic hierarchy process
AN: Anorexia nervosa
AQ-10: Autism spectrum quotient-10
BLRT: Bootstrapped likelihood ratio test
BMI: Body mass index
D: Duration
DASS: Depression anxiety stress scale
DSM-5: Diagnostic and statistical manual of mental disorders version 5
ED: Eating disorder
EDE-Q: Eating disorder examination questionnaire
GAD: Generalised anxiety disorder
LMM: Linear mixed model
LPA: Latent profile analysis
MDD: Major depressive disorder
OCD: Obsessive-compulsive disorder
OCI: Obsessive compulsive inventory
S: Severity
SD: Standard deviation
SE-AN: Severe and enduring anorexia nervosa
SL-AN: Severe and longstanding anorexia nervosa
WSAS: Work and social adjustment scale
