# Supplementary material for: A person-centred and data-driven approach to phenotyping anorexia nervosa
Source: J Eat Disord. 2026 May 19;14:163. doi: 10.1186/s40337-026-01632-8 (PMC13366680; doi:10.1186/s40337-026-01632-8)
Supplement: Supplementary file 1 — Supplementary Material 1. [file 40337_2026_1632_MOESM1_ESM.docx]

**Supplementary Material I**

**Contents**

[**1. Figures** 1](#_Toc221113331)

[**2. Tables** 5](#_Toc221113333)

# **1. Figures**

# **
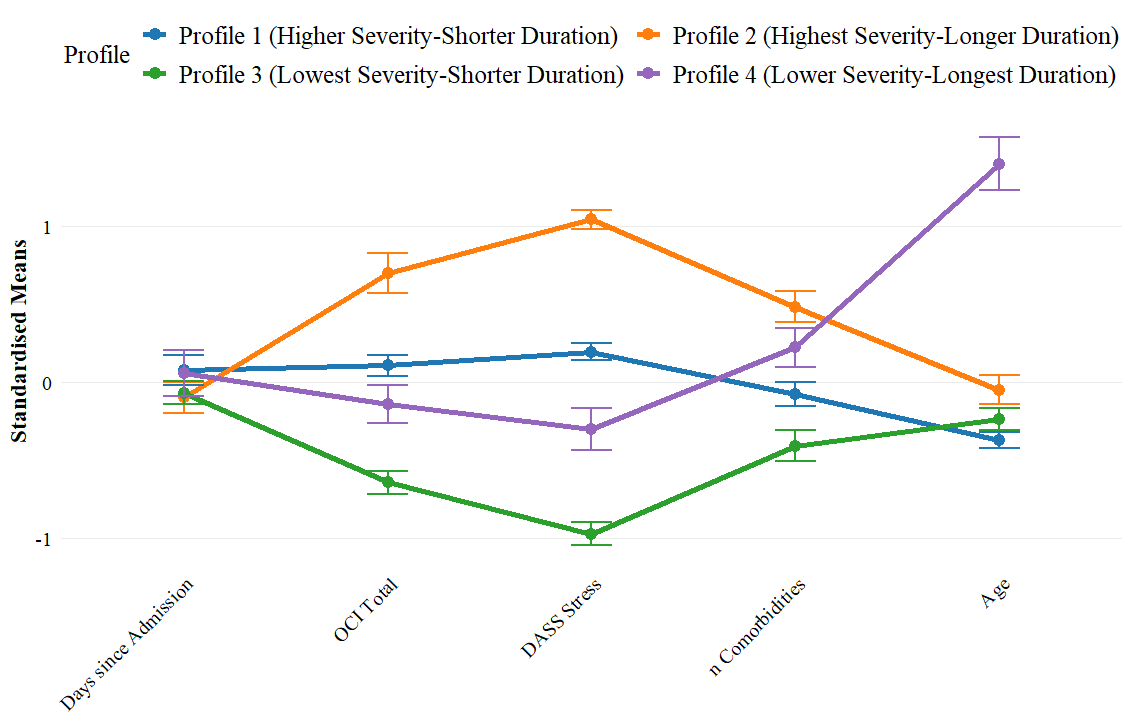
**

**Figure S1. Standardised Means of the Variables not included in the Latent Profile Analysis, per Profile**

Note. This line graph displays the standardised means of each profile across the variables not included in the latent profile analysis. The values shown represent standardised (relative) means rather than raw means, allowing for direct comparison between profiles. The lines connecting the points are included purely for visual clarity to highlight patterns and differences between profiles and should not be interpreted as indicating a progression over time or any causal relationship. Error bars were calculated using standard error. Days since admission refers to the number of days that have passed between admission to treatment and baseline assessment. Abbreviations: DASS = Depression Anxiety Stress scale, n = number of, OCI = Obsessive Compulsive Inventory


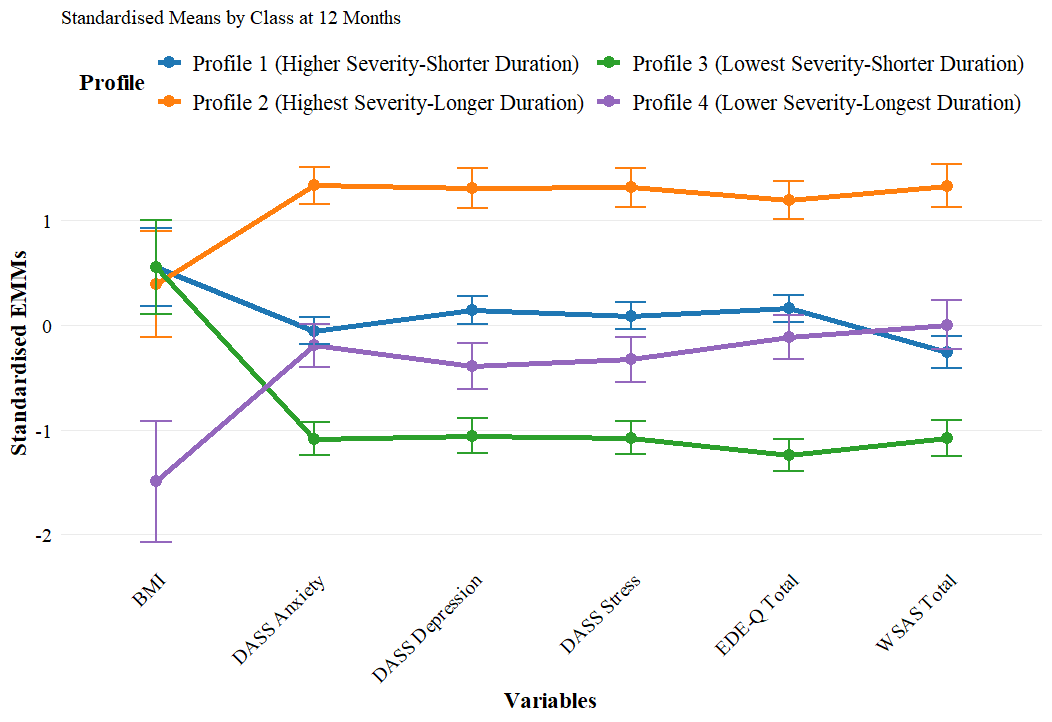


**Figure S2. Standardised EMMs of all Variables at the 12-month Follow-up**

Note. This line graph displays the standardised EMMs of each profile across the variables at the 12-month follow-up time-point. The values shown represent standardised (relative) means rather than raw means, allowing for direct comparison between profiles. The lines connecting the points are included purely for visual clarity to highlight patterns and differences between profiles and should not be interpreted as indicating a progression over time or any causal relationship. Error bars were calculated using standard error. Abbreviations: BMI = Body Mass Index, DASS = Depression Anxiety Stress Scale, EDE-Q = Eating Disorder Examination Questionnaire, EMMs = Estimated Marginal Means, WSAS = Work and Social Adjustment Questionnaire


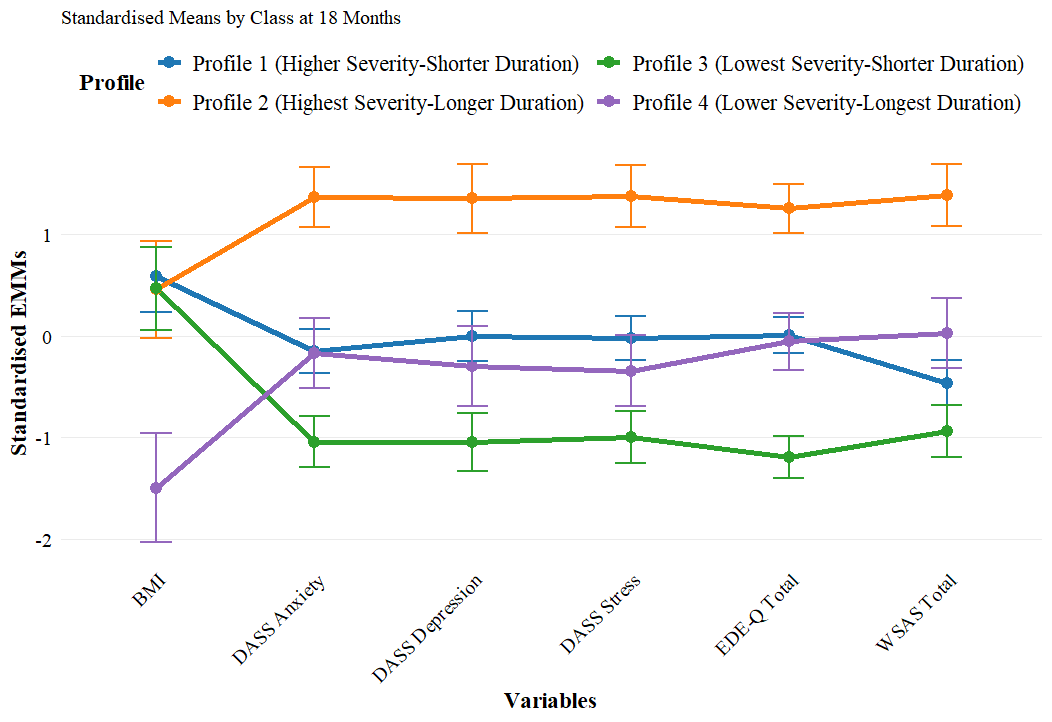


**Figure S3. Standardised EMMs of all Variables at the 18-month Follow-up**

Note. This line graph displays the standardised EMMs of each profile across the variables at the 18-month follow-up time-point. The values shown represent standardised (relative) means rather than raw means, allowing for direct comparison between profiles. The lines connecting the points are included purely for visual clarity to highlight patterns and differences between profiles and should not be interpreted as indicating a progression over time or any causal relationship. Error bars were calculated using standard error. Abbreviations: BMI = Body Mass Index, DASS = Depression Anxiety Stress Scale, EDE-Q = Eating Disorder Examination Questionnaire, EMMs = Estimated Marginal Means, WSAS = Work and Social Adjustment Questionnaire

# **2. Tables**

**Table S1. Number of Missing Baseline Data Points per Variable: Total Sample and per Profile**

| **Variable** | **Total Sample** | **Higher S-Shorter D** | **Highest S-Longer D** | **Lowest S-Shorter D** | **Lower S-Longest D** |  |
| --- | --- | --- | --- | --- | --- | --- |
| Treatment Type | 0 | 0 | 0 | 0 | 0 | |
| Days since Adm.* | 0 | 0 | 0 | 0 | 0 | |
| Age | 12 | 8 | 1 | 1 | 2 | |
| Sex | 0 | 0 | 0 | 0 | 0 | |
| BMI | 33 | 12 | 3 | 10 | 8 | |
| Ethnicity | 0 | 0 | 0 | 0 | 0 | |
| Illness Duration | 16 | 10 | 1 | 3 | 2 | |
| Age at onset | 16 | 10 | 1 | 3 | 2 | |
| n Comorbidities | 0 | 0 | 0 | 0 | 0 | |
| Diag. Depression | 0 | 0 | 0 | 0 | 0 | |
| Diag. Anxiety | 0 | 0 | 0 | 0 | 0 | |
| Diag. OCD | 0 | 0 | 0 | 0 | 0 | |
| Diag. ADHD | 0 | 0 | 0 | 0 | 0 | |
| Diag. Autism | 0 | 0 | 0 | 0 | 0 | |
| Diag. PD | 0 | 0 | 0 | 0 | 0 | |
| AQ-10 | 0 | 0 | 0 | 0 | 0 | |
| DASS Anxiety | 6 | 2 | 1 | 3 | 0 | |
| DASS Depression | 6 | 2 | 1 | 3 | 0 | |
| DASS Stress | 6 | 2 | 1 | 3 | 0 | |
| EDE-Q | 9 | 4 | 2 | 3 | 0 | |
| OCI | 7 | 3 | 1 | 2 | 1 | |
| WSAS | 4 | 1 | 0 | 3 | 0 | |

Note. *Days since admission refers to the number of days that passed between admission to treatment and baseline measurement. Abbreviations: ADHD = Attention-Deficit/Hyperactivity Disorder, Adm. = admission, D = Duration, DASS = Depression Anxiety Stress Scale, Diag. = Lifetime Diagnosis, EDE-Q = Eating Disorder Examination Questionnaire, Highest S-Longer D = Highest S-Longer D, IQR= interquartile range, OCD = Obsessive Compulsive Disorder, OCI = Obsessive Compulsive Index, med= median, PD = Panic Disorder, S = Severity, WSAS = Work and Social Adjustment Scale

Table S2. Model Fit Indices and Mean Posterior Probabilities of the Latent Profile Analysis

| **Model** | **AIC** | **BIC** | **Entropy** | **Mean PP** |
| --- | --- | --- | --- | --- |
| 1 Profile | 7,609.47 | 7,664.71 | Inf | 1.00 |
| 2 Profiles | 7,030.62 | 7,145.04 | 0.82 | **0.95** |
| 3 Profiles | 6,903.39 | 7,076.99 | 0.78 | 0.91 |
| 4 Profiles | 6,755.64 | **6,988.42** | 0.78 | 0.89 |
| 5 Profiles | 6,728.43 | 7,020.39 | 0.78 | 0.87 |
| 6 Profiles | **6,686.13** | 7,037.27 | 0.77 | 0.85 |

Note. Statistics indicating the best model fit are printed in bold. Models with one profile have perfect entropy and mean PP, as individuals can only be assigned to one profile. Abbreviations: AIC = Akaike-Information Criterion, BIC = Bayesian Information Criterion, Inf = infinitive, PP = Posterior Probabilities

**Table S3. Kruskal-Wallis tests and Dunn’s Post-Hoc tests of Profile Differences: Variables included in the Latent Profile Analysis**

| **Variable** | **Test** | **Test-statistic** | **η^2^** | **g** |
| --- | --- | --- | --- | --- |
| Illness Duration | Kruskal-Wallis*** | H(3) = 141.79 | 0.38 |  |
|  | Higher S-Shorter D vs Seve-Long*** |  |  | -0.96 |
|  | Higher S-Shorter D vs Lowest S-Shorter D |  |  | -0.18 |
|  | Higher S-Shorter D vs Lower S-Longest D*** |  |  | -2.74 |
|  | Highest S-Longer D vs Lowest S-Shorter D** |  |  | 0.74 |
|  | Highest S-Longer D vs Lower S-Longest D*** |  |  | -1.41 |
|  | Lowest S-Shorter D vs Lower S-Longest D*** |  |  | -2.32 |
|  |  |  |  |  |
| WSAS Total | Kruskal-Wallis*** | H(3) = 121.46 | 0.31 |  |
|  | Higher S-Shorter D vs Highest S-Longer D*** |  |  | -0.96 |
|  | Higher S-Shorter D vs Lowest S-Shorter D*** |  |  | 1.11 |
|  | Higher S-Shorter D vs Lower S-Longest D |  |  | 0.00 |
|  | Highest S-Longer D vs Lowest S-Shorter D*** |  |  | 2.13 |
|  | Highest S-Longer D vs Lower S-Longest D*** |  |  | 0.92 |
|  | Lowest S-Shorter D vs Lower S-Longest D*** |  |  | -1.02 |
|  |  |  |  |  |
| DASS Depression | Kruskal-Wallis*** | H(3) = 235.9 | 0.62 |  |
|  | Higher S-Shorter D vs Highest S-Longer D*** |  |  | -1.62 |
|  | Higher S-Shorter D vs Mod-Full Stage*** |  |  | 2.04 |
|  | Higher S-Shorter D vs Mod-Long*** |  |  | 1.17 |
|  | Highest S-Longer D vs Mod-Full Stage** |  |  | 4.15 |
|  | Highest S-Longer D vs Mod-Long*** |  |  | 2.86 |
|  | Lowest S-Shorter D vs Lower S-Longest D* |  |  | -0.68 |
|  |  |  |  |  |
| DASS Anxiety | Kruskal-Wallis*** | H(3) = 209.41 | 0.55 |  |
|  | Higher S-Shorter D vs Highest S-Longer D*** |  |  | -1.83 |
|  | Higher S-Shorter D vs Lowest S-Shorter D*** |  |  | 1.87 |
|  | Higher S-Shorter D vs Lower S-Longest D |  |  | 0.33 |
|  | Highest S-Longer D vs Lowest S-Shorter D*** |  |  | 3.54 |
|  | Highest S-Longer D vs Lower S-Longest D*** |  |  | 1.65 |
|  | Lowest S-Shorter D vs Lower S-Longest D*** |  |  | -1.10 |
|  |  |  |  |  |
| EDE-Q total | Kruskal-Wallis*** | H(3) = 128.9 | 0.34 |  |
|  | Higher S-Shorter D vs Highest S-Longer D*** |  |  | -0.98 |
|  | Higher S-Shorter D vs Lowest S-Shorter D*** |  |  | 1.54 |
|  | Higher S-Shorter D vs Lower S-Longest D*** |  |  | 0.54 |
|  | Highest S-Longer D vs Lowest S-Shorter D*** |  |  | 2.11 |
|  | Highest S-Longer D vs Lower S-Longest D*** |  |  | 1.17 |
|  | Lowest S-Shorter D vs Lower S-Longest D*** |  |  | -0.74 |
|  |  |  |  |  |
| AQ-10 Total | Kruskal-Wallis*** | H(3) = 67.6 | 0.17 |  |
|  | Higher S-Shorter D vs Highest S-Longer D |  |  | -0.36 |
|  | Higher S-Shorter D vs Lowest S-Shorter D*** |  |  | 0.92 |
|  | Higher S-Shorter D vs Lower S-Longest D* |  |  | 0.49 |
|  | Highest S-Longer D vs Lowest S-Shorter D*** |  |  | 1.27 |
|  | Highest S-Longer D vs Lower S-Longest D*** |  |  | 0.81 |
|  | Lowest S-Shorter D vs Lower S-Longest D |  |  | -0.41 |
|  |  |  |  |  |
| BMI | Kruskal-Wallis | H(3) = 4.75 | 0.01 |  |

Note. Kruskal-Wallis tests were deemed significant below a Bonferroni-corrected α = 0.007 threshold. Post-hoc tests returned multiple-comparison corrected p-values and were considered significant at *α* = 0.05. Abbreviations: AQ-10 = Autism Spectrum Quotient, D = Duration, DASS = Depression Anxiety Stress Scale, g = Hedge’s g, EDE-Q = Eating Disorder Examination Questionnaire, η^2^ = eta squared, S = Severity, WSAS = Work and Social Adjustment Scale; **p-value* < 0.05, ***p-value ****< 0.01, *p-value* < 0.001

**Table S4. Kruskal-Wallis tests and Dunn’s Post-Hoc tests of Profile Differences: Variables not included in the Latent Profile Analysis**

| **Variable** | **Test** | **Test-statistic** | **η^2^** | **g** |
| --- | --- | --- | --- | --- |
| DASS Stress | Kruskal-Wallis*** | H((3) = 186.6 | 0.49 |  |
|  | Higher S-Shorter D vs Highest S-Longer D*** |  |  | -1.35 |
|  | Higher S-Shorter D vs Lowest S-Shorter D*** |  |  | 1.70 |
|  | Higher S-Shorter D vs Lower S-Longest D* |  |  | 0.63 |
|  | Highest S-Longer D vs Lowest S-Shorter D*** |  |  | 3.17 |
|  | Highest S-Longer D vs Lower S-Longest D*** |  |  | 1.74 |
|  | Lowest S-Shorter D vs Lower S-Longest D*** |  |  | -0.81 |
|  |  |  |  |  |
| OCI Total | Kruskal-Wallis*** | H(3) = 77.1 | 0.20 |  |
|  | Higher S-Shorter D vs Highest S-Longer D* |  |  | -0.62 |
|  | Higher S-Shorter D vs Lowest S-Shorter D*** |  |  | 0.96 |
|  | Higher S-Shorter D vs Lower S-Longest D |  |  | 0.29 |
|  | Highest S-Longer D vs Lowest S-Shorter D*** |  |  | 1.43 |
|  | Highest S-Longer D vs Lower S-Longest D*** |  |  | 0.79 |
|  | Lowest S-Shorter D vs Lower S-Longest D** |  |  | -0.63 |
|  |  |  |  |  |
| Age | Kruskal-Wallis*** | H(3) = 96.64 | 0.25 |  |
|  | Higher S-Shorter D vs Highest S-Longer D* |  |  | -0.47 |
|  | Higher S-Shorter D vs Lowest S-Shorter D |  |  | -0.20 |
|  | Higher S-Shorter D vs Lower S-Longest D*** |  |  | -2.09 |
|  | Highest S-Longer D vs Lowest S-Shorter D |  |  | 0.25 |
|  | Highest S-Longer D vs Lower S-Longest D*** |  |  | -1.40 |
|  | Lowest S-Shorter D vs Lower S-Longest D*** |  |  | -1.72 |
|  |  |  |  |  |
| n Comorbidities | Kruskal-Wallis*** | H(3) = 42.31 | 0.10 |  |
|  | Higher S-Shorter D vs Highest S-Longer D*** |  |  | -0.60 |
|  | Higher S-Shorter D vs Lowest S-Shorter D* |  |  | 0.34 |
|  | Higher S-Shorter D vs Lower S-Longest D |  |  | -0.31 |
|  | Highest S-Longer D vs Lowest S-Shorter D*** |  |  | 0.92 |
|  | Highest S-Longer D vs Lower S-Longest D |  |  | 0.28 |
|  | Lowest S-Shorter D vs Lower S-Longest D*** |  |  | -0.64 |
|  |  |  |  |  |
| Days since admission | Kruskal-Wallis | H(3) = 2.95 | 0.00 |  |

Note. Days since admission refers to the number of days that passed between admission to treatment and baseline measurement. Kruskal-Wallis tests were deemed significant below a Bonferroni-corrected *α* = 0.0045 threshold. Post-hoc tests returned multiple-comparison corrected p-values and were considered significant at *α* = 0.05. Abbreviations: D = duration, DASS=Depression Anxiety Stress Scale, g = Hedge’s g, η^2^ = eta squared, OCI = Obsessive Compulsive Index S = Severity; **p-value* < 0.05, ***p-value* < 0.01, ****p-value* < 0.001

**Table S5. Estimated Marginal Means and their Standard Errors next to Raw Means and their Standard Deviations at the 12- and 18-month Follow-up Time-points**

| **Variable** | **Profile** | **EMM (SE)** | **Raw Mean (SD)** |
| --- | --- | --- | --- |
| *12 months* |  |  |  |
| BMI | Higher S-Shorter D | 17.03 (0.24) | 17.66 (2.22) |
|  | Highest S-Longer D | 16.92 (0.33) | 17.31 (3.41) |
|  | Lowest S-Shorter D | 17.03 (0.29) | 16.94 (2.36) |
|  | Lower S-Longest D | 15.71 (0.37) | 16.32 (2.67) |
| Depression | Higher S-Shorter D | 23.83 (0.88) | 24.22 (10.93) |
|  | Highest S-Longer D | 31.26 (1.21) | 32.33 (9.21) |
|  | Lowest S-Shorter D | 16.17 (1.04) | 17.25 (11.03) |
|  | Lower S-Longest D | 20.42 (1.41) | 21.25 (11.50) |
| Anxiety | Higher S-Shorter D | 14.12 (0.71) | 13.51 (7.62) |
|  | Highest S-Longer D | 21.76 (0.97) | 21.74 (9.32) |
|  | Lowest S-Shorter D | 8.46 (0.84) | 9.12 (7.63) |
|  | Lower S-Longest D | 13.36 (1.14) | 13.00 (10.16) |
| Stress | Higher S-Shorter D | 23.65 (0.70) | 23.37 (9.02) |
|  | Highest S-Longer D | 30.10 (0.96) | 30.75 (7.30) |
|  | Lowest S-Shorter D | 17.50 (0.83) | 18.34 (8.89) |
|  | Lower S-Longest D | 21.46 (1.13) | 21.90 (9.00) |
| EDE-Q | Higher S-Shorter D | 3.56 (0.11) | 3.58 (1.28) |
|  | Highest S-Longer D | 4.41 (0.14) | 4.32 (1.17) |
|  | Lowest S-Shorter D | 2.43 (0.13) | 2.51 (1.41) |
|  | Lower S-Longest D | 3.34 (0.17) | 3.35 (1.69) |
| WSAS | Higher S-Shorter D | 17.92 (0.81) | 16.88 (10.00) |
|  | Highest S-Longer D | 26.38 (1.11) | 25.68 (10.82) |
|  | Lowest S-Shorter D | 13.55 (0.92) | 13.58 (10.67) |
|  | Lower S-Longest D | 19.30 (1.25) | 18.79 (12.71) |
| *18 months* |  |  |  |
| BMI | Higher S-Shorter D | 17.88 (0.28) | 17.73 (2.54) |
|  | Highest S-Longer D | 17.78 (0.39) | 16.93 (2.73) |
|  | Lowest S-Shorter D | 17.79 (0.33) | 17.67 (2.78) |
|  | Lower S-Longest D | 16.20 (0.44) | 16.50 (2.76) |
| Depression | Higher S-Shorter D | 21.76 (1.19) | 22.45 (12.19) |
|  | Highest S-Longer D | 28.33 (1.65) | 29.74 (12.16) |
|  | Lowest S-Shorter D | 16.69 (1.40) | 14.73 (10.07) |
|  | Lower S-Longest D | 20.32 (1.91) | 18.82 (12.64) |
| Anxiety | Higher S-Shorter D | 12.79 (0.96) | 13.86 (8.38) |
|  | Highest S-Longer D | 19.57 (1.32) | 21.28 (11.68) |
|  | Lowest S-Shorter D | 8.80 (1.13) | 8.54 (9.24) |
|  | Lower S-Longest D | 12.72 (1.54) | 13.65 (10.85) |
| Stress | Higher S-Shorter D | 21.92 (0.97) | 22.88 (9.52) |
|  | Highest S-Longer D | 28.14 (1.34) | 29.49 (8.88) |
|  | Lowest S-Shorter D | 17.56 (1.14) | 17.27 (7.78) |
|  | Lower S-Longest D | 20.48 (1.57) | 19.59 (12.13) |
| EDE-Q | Higher S-Shorter D | 3.29 (0.14) | 3.49 (1.16) |
|  | Highest S-Longer D | 4.24 (0.18) | 4.56 (1.06) |
|  | Lowest S-Shorter D | 2.38 (0.16) | 2.37 (1.29) |
|  | Lower S-Longest D | 3.25 (0.21) | 3.27 (1.58) |
| WSAS | Higher S-Shorter D | 14.58 (1.13) | 15.71 (9.82) |
|  | Highest S-Longer D | 23.90 (1.56) | 25.21 (11.07) |
|  | Lowest S-Shorter D | 12.19 (1.27) | 12.18 (9.91) |
|  | Lower S-Longest D | 17.04 (1.74) | 17.12 (12.42) |

Note. To ensure transparency, this table presents both the estimated marginal means and the raw means of each variable at the 12- and 18-month time points. Abbreviations: BMI = Body Mass Index, D = Duration, DASS = Depression Anxiety Stress Scale, EDE-Q = Eating Disorder Examination Questionnaire, EMM = Estimated Marginal Means, SD = Standard Deviations, S = Severity, SE = Standard Error, WSAS = Work and Social Adjustment Scale

**Table S6. EMMs and SEs on the Outcome Variables per Profile compared to Population-normed Thresholds of “Severe” – 12 months**

| **Variable** | **Higher S-Shorter D** | **Highest S-Longer D** | | **Lowest S-Shorter D** | **Lower S-Longest D** | **Literature-derived Thresholds** | | | |
| --- | --- | --- | --- | --- | --- | --- | --- | --- | --- |
|  |  |  |  |  |  | **Mild** | **Moderate** | **Severe** | **Extreme** |
| BMI | 17.03 ± 0.24 | | 16.92 ± 0.33 | 17.03 ± 0.29 | 15.71 ± 37 | ≥ 17.0 | 16-16.99 | 15-15.99 | < 15 |
| DASS Depression | **23.83** ± 0.88 | | **31.26** ± 1.21 | 16.17 ± 1.04 | 20.42 ± 1.41 | 10-13 | 14-20 | 21-27 | >27 |
| DASS Anxiety | 14.12 ± 0.71 | | **21.76** ± 0.97 | 8.46 ± 0.84 | 13.36 ± 1.14 | 8-9 | 10-14 | 15-19 | >19 |
| DASS Stress | 23.65 ± 0.70 | | **30.10** ± 0.96 | 17.50 ± 0.83 | 21.46 ± 1.13 | 15-18 | 19-25 | 26-33 | >33 |
| EDE-Q | 3.56 ± 0.11 | | **4.41** ± 0.14 | 2.43 ± 0.13 | 3.34 ± 0.17 | NA | NA | 4 | NA |
| WSAS | 17.92 ± 0.81 | | **26.38** ± 1.11 | 13.55 ± 0.92 | 19.30 ± 1.25 | <9 | 10-20 | >20 | NA |

Note. EMMs that cross the “severe” threshold are printed in bold. Cut-off definitions: BMI,(6) DASS subscales,(7) EDE-Q,(8) WSAS.(9) Abbreviations: BMI = Body Mass Index, D = Duration, DASS = Depression Anxiety Stress Scale, EDE-Q = Eating Disorder Examination Questionnaire, EMMs = Estimated Marginal Means, Mod = Moderate, S = Severity, SE = Standard Error, WSAS = Work and Social Adjustment Scale

**Table S7. EMMs and SEs on the Outcome Variables per Profile compared to Population-normed Thresholds of “Severe” – 18 months**

| **Variable** | **Higher S-Shorter D** | **Highest S-Longer D** | | **Lowest S-Shorter D** | **Lower S-Longest D** | **Literature-derived Thresholds** | | | |
| --- | --- | --- | --- | --- | --- | --- | --- | --- | --- |
|  |  |  |  |  |  | **Mild** | **Moderate** | **Severe** | **Extreme** |
| BMI | 17.88 ± 0.28 | | 17.78 ± 0.39 | 17.79 ± 0.33 | 16.20 ± 0.44 | ≥ 17.0 | 16-16.99 | 15-15.99 | < 15 |
| DASS Depression | **21.76** ± 1.19 | | **28.33** ± 1.65 | 16.69 ± 1.40 | 20.32 ± 1.91 | 10-13 | 14-20 | 21-27 | >27 |
| DASS Anxiety | 12.79 ± 0.96 | | **19.57** ± 1.32 | 8.80 ± 1.13 | 12.72 ± 1.54 | 8-9 | 10-14 | 15-19 | >19 |
| DASS Stress | 21.92 ± 0.97 | | **28.14** ± 1.34 | 17.56 ± 1.14 | 20.48 ± 1.57 | 15-18 | 19-25 | 26-33 | >33 |
| EDE-Q | 3.29 ± 0.14 | | **4.24** ± 0.18 | 2.38 ± 0.16 | 3.25 ± 0.21 | NA | NA | 4 | NA |
| WSAS | 14.58 ± 1.13 | | **23.90** ± 1.56 | 12.19 ± 1.27 | 17.04 ± 1.74 | <9 | 10-20 | >20 | NA |

Note. EMMs that cross the “severe” threshold are printed in bold. Cut-off definitions: BMI,(6) DASS subscales,(7) EDE-Q,(8) WSAS.(9) Abbreviations: BMI = Body Mass Index, D = Duration, DASS = Depression Anxiety Stress Scale, EDE-Q = Eating Disorder Examination Questionnaire, EMMs = Estimated Marginal Means, Mod = Moderate, S = Severity, SE = Standard Error, WSAS = Work and Social Adjustment Scale

**Table S8. Main and Interaction Effects of the LMMs of all Outcome Variables**

| **Outcome** | **Time** | **p** | **Profile** | **p** | **Time x Profile Interaction** | **p** |
| --- | --- | --- | --- | --- | --- | --- |
| **BMI** | F(1,1157.99) = 182.84 | **<0.001** | F(3,414.62) = 0.91 | 0.44 | F(3,1157.90) = 2.05 | 0.11 |
| **DASS Depression** | F(1,293.59) = 18.89 | **<0.001** | F(3,376.71) = 117.66 | **<0.001** | F(3,293.73) = 10.40 | **<0.001** |
| **DASS Anxiety** | F(1,291.92) = 19.22 | **<0.001** | F(3,373.86) = 109.55 | **<0.001** | F(3,292.34) = 7.03 | **<0.001** |
| **DASS Stress** | F(1,286.97) = 24.47 | **<0.001** | F(3,376.81) = 100.84 | **<0.001** | F(3,287.37) = 4.99 | **0.002** |
| **EDE-Q** | F(1,264.49) = 27.23 | **<0.001** | F(3,378.25) = 65.33 | **<0.001** | F(3,265.01) = 4.37 | **0.005** |
| **WSAS** | F(1,272.03) = 85.13 | **<0.001** | F(3,380.90) = 62.35 | **<0.001** | F(3,271.63) = 3.60 | **0.01** |

Note. Significant p-values are printed in bold. Abbreviations: BMI = Body Mass Index, DASS = Depression Anxiety Stress Scale, EDE-Q = Eating Disorder Examination Questionnaire, LMM = Linear Mixed Model, p = p-value, WSAS = Work and Social Adjustment Scale

**Table S9.** **Estimated LMM Slopes for All Outcome Variables by Profile**

|  | **Higher S-Shorter D** | | **Highest S-Longer D** | | **Lowest S-Shorter D** | | **Lower S-Longest D** | |
| --- | --- | --- | --- | --- | --- | --- | --- | --- |
| **Outcome** | **B (95% CI)** | **p** | **B (95% CI)** | **p** | **B (95% CI)** | **p** | **B (95% CI)** | **p** |
| **BMI** | 0.14 (0.11–0.17) | **<0.001** | 0.14 (0.10–0.18) | **<0.001** | 0.13 (0.09–0.16) | **< 0.001** | 0.08 (0.04–0.12) | **<0.001** |
| **DASS Anxiety** | -0.22 (-0.33–-0.11) | **<0.001** | -0.36 (-0.51–-0.22) | **<0.001** | 0.06 (-0.07–0.18) | 0.38 | -0.11 (-0.28–0.06) | 0.22 |
| **DASS Depression** | -0.35 (-0.47–-0.22) | **<0.001** | -0.49 (-0.67–-0.31) | **<0.001** | 0.09 (-0.07–0.24) | 0.27 | -0.02 (-0.22–0.19) | 0.88 |
| **DASS Stress** | -0.29 (-0.40–-0.18) | **<0.001** | -0.33 (-0.48–-0.17) | **<0.001** | 0.01 (-0.12–0.14) | 0.87 | -0.16 (-0.34–0.02) | 0.07 |
| **EDE-Q** | -0.05 (-0.06–-0.03) | **<0.001** | -0.02 (-0.04–0.00) | 0.08 | -0.01 (-0.03–0.01) | 0.18 | -0.02 (-0.04–0.00) | 0.10 |
| **WSAS** | -0.56 (-0.69–-0.42) | **<0.001** | -0.41 (-0.60–-0.23) | **<0.001** | 0.13 (0.09–0.16) | **<0.001** | -0.38 (-0.58–-0.17) | **<0.001** |

Note. Significant p-values are printed in bold. Abbreviations: B = Unstandardised Beta, BMI = Body Mass Index, D = Duration, DASS = Depression Anxiety Stress Scale, EDE-Q = Eating Disorder Examination Questionnaire, LMM = Linear Mixed Model, p = p-value, S = Severity, WSAS = Work and Social Adjustment Scale
