# Supplementary material for: A person-centred and data-driven approach to phenotyping anorexia nervosa
Source: J Eat Disord. 2026 May 19;14:163. doi: 10.1186/s40337-026-01632-8 (PMC13366680; doi:10.1186/s40337-026-01632-8)
Supplement: Supplementary file 2 — Supplementary Material 2. [file 40337_2026_1632_MOESM2_ESM.docx]

**Supplementary Material II**

**Contents**

[**1. Latent Profile Analysis Procedure** 1](#_Toc221694720)

[**2. Residual Correlations of the Chi-Square Statistics** 2](#_Toc221694721)

[**3. References** 5](#_Toc221694722)

# **1. Latent Profile Analysis Procedure**

Latent profile analysis (LPA) was carried out by estimating several models with different numbers of profiles, varying from one to six. The upper limit of six profiles was chosen because simpler models are generally preferred(1). However, if the six-profile solution had demonstrated the best fit, additional models with more than six profiles would have been tested to ensure no higher-profile solution provided a superior fit. As latent profile analyses cannot be performed with missing data, missing values were imputed using predictive mean matching. All variables included in the LPA, illness duration, body mass index, Eating Disorder Examination Questionnaire global score, Work and Social Adjustment Scale total score, Depression Anxiety Stress Scale depression and anxiety subscale scores, and Autism Spectrum Quotient-10 total score, were used as predictors. This method was selected because it generates realistic values and preserves the original distributional properties of the data(2).

Model fit was evaluated using multiple indices, including the Bayesian Information Criterion (BIC), Akaike Information Criterion (AIC), mean posterior probabilities (PP), entropy, and the Analytic Hierarchy Process (AHP). Both AIC and BIC assess the trade-off between model fit and complexity, with values increasing when the improvement in fit from adding an additional profile does not justify the increase in model complexity(3,4). Mean PP reflects the probability of individuals belonging to their assigned class; a value of 1 denotes complete certainty in classification(3,4). Entropy measures the separation between latent profiles, with higher values indicating greater distinction and lower classification uncertainty(3,4). The AHP is a multi-criteria decision-making approach that integrates several fit indices, AIC, Approximate Weight of Evidence, BIC, Classification Likelihood Criterion, and Kullback Information Criterion, to generate a comprehensive ranking of model solutions. This method provides a more holistic assessment than any single index(5). Importantly, these indices are not weighted equally: BIC is generally considered more robust and reliable than AIC, and both are typically prioritised over entropy or mean PP(5). The AHP received the greatest weight in the final evaluation(5). The Bootstrapped Likelihood Ratio Test (BLRT), which evaluates the relative fit of a model with *k* profiles compared to one with *k - 1* profiles, was not conducted due to methodological constraints. Specifically, the variables were standardised prior to the latent profile analysis, and the available R implementation of the BLRT does not accommodate standardised input variables.

# **2. Residual Correlations of the Chi-Square Statistics**

Standardised residual correlations were considered to examine how the different profiles contributed to the chi-square statistics of profile differences regarding lifetime MDD and GAD diagnoses. Higher- or lower-than-expected proportions indicate that the observed counts significantly deviated from the expected counts under the assumption of no association between profile membership and lifetime diagnosis. For MDD, standardised residual correlations indicated that *Highest Severity-Longer Duration* and *Lower Severity-Longest Duration* had fewer individuals without the diagnosis than expected; however, only *Highest Severity-Longer Duration* showed a significantly higher-than-expected proportion of individuals with MDD. Conversely*, Lowest Severity-Shorter Duration* had significantly more individuals without, and fewer with, a diagnosis than expected (Figure S4.1). For GAD, *Highest Severity-Longer Duration* again exhibited a significantly higher-than-expected proportion of individuals with the diagnosis, and fewer without, while *Lowest Severity-Shorter Duration* showed the opposite pattern, significantly fewer individuals with GAD and more without than expected (Figure S4.2).


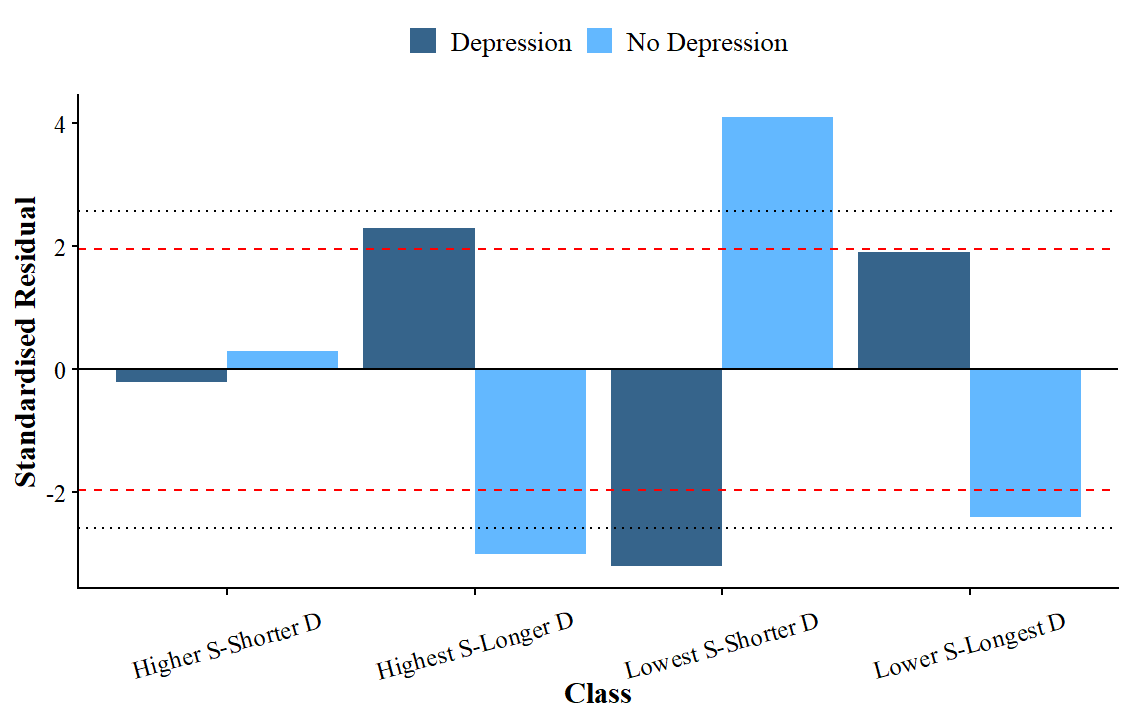


**Figure SII.2.1. Standardised Residual Correlations of the Chi-Square test examining Differences in Lifetime Major Depressive Disorder Diagnoses between Profiles**

Note. The dashed red line indicates the residual correlation is significant at *p* < 0.05; the dotted blue line indicates a significance level of *p* < 0.01. Abbreviations: D = duration, S = Severity


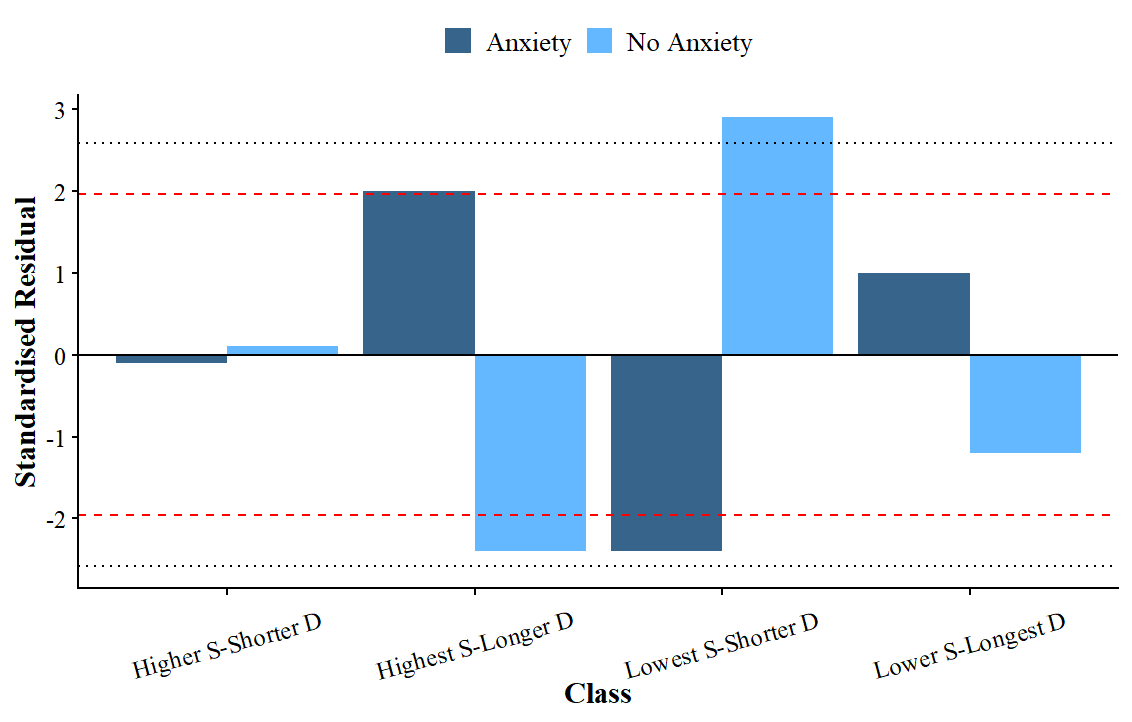


**Figure SII.2.2. Standardised Residual Correlations of the Chi-Square test examining Differences in Lifetime Generalised Anxiety Disorder Diagnoses between Profiles**

Note. The dashed red line indicates the residual correlation is significant at *p* < 0.05; the dotted blue line indicates a significance level of *p* < 0.01. Abbreviations: D = duration, S = Severity

# **3. References**

1. Spurk D, Hirschi A, Wang M, Valero D, Kauffeld S. Latent profile analysis: A review and “how to” guide of its application within vocational behavior research. J Vocat Behav. 2020 Aug 1;120:103445.

2. Allison P. Imputation by Predictive Mean Matching: Promise & Peril [Internet]. Statistical Horizons. 2015 [cited 2025 Aug 13]. Available from: https://statisticalhorizons.com/predictive-mean-matching/

3. Băjenaru L, Balog A, Dobre C, Drăghici R, Prada GI. Latent profile analysis for quality of life in older patients. BMC Geriatr. 2022 Dec;22(1):1–7.

4. Schwarz G. Estimating the Dimension of a Model. Ann Stat. 1978 Mar;6(2):461–4.

5. Akogul S, Erişoğlu M. An Approach for Determining the Number of Clusters in a Model-Based Cluster Analysis. Entropy. 2017 Aug 29;19:452.

6. American Psychiatric Association. Diagnostic and Statistical Manual of Mental Disorders [Internet]. Fifth Edition. American Psychiatric Association; 2013 [cited 2025 Aug 5]. Available from: https://psychiatryonline.org/doi/book/10.1176/appi.books.9780890425596

7. Lovibond PF, Lovibond SH. The structure of negative emotional states: comparison of the Depression Anxiety Stress Scales (DASS) with the Beck Depression and Anxiety Inventories. Behav Res Ther. 1995 Mar;33(3):335–43.

8. Dahlgren CL, Stedal K, Rø Ø. Eating Disorder Examination Questionnaire (EDE-Q) and Clinical Impairment Assessment (CIA): clinical norms and functional impairment in male and female adults with eating disorders. Nord J Psychiatry. 2017 May 19;71(4):256–61.

9. Mundt JC, Marks IM, Shear MK, Greist JM. The Work and Social Adjustment Scale: a simple measure of impairment in functioning. Br J Psychiatry. 2002 May;180(5):461–4.
